# Chlorophyll and carbohydrate metabolism in developing silique and seed are prerequisite to seed oil content of *Brassica napus* L.

**DOI:** 10.1186/1999-3110-55-34

**Published:** 2014-03-19

**Authors:** Shuijin Hua, Zhong-Hua Chen, Yaofeng Zhang, Huasheng Yu, Baogang Lin, Dongqing Zhang

**Affiliations:** 1 Institute of Crop and Nuclear Technology Utilization, Zhejiang Academy of Agricultural Sciences, Hangzhou, 310021 P.R. China; 2 School of Science and Health, University of Western Sydney, Penrith, 2751NSW Australia

**Keywords:** Biomass, *Brassica napus* L, Carbohydrate, Chlorophyll content, Seed oil content

## Abstract

**Background:**

Although the seed oil content in canola is a crucial quality determining trait, the regulatory mechanisms of its formation are not fully discovered. This study compared the silique and seed physiological characteristics including fresh and dry weight, seed oil content, chlorophyll content, and carbohydrate content in a high oil content line (HOCL) and a low oil content line (LOCL) of canola derived from a recombinant inbred line in 2010, 2011, and 2012. The aim of the investigation is to uncover the physiological regulation of silique and seed developmental events on seed oil content in canola.

**Results:**

On average, 83% and 86% of silique matter while 69% and 63% of seed matter was produced before 30 days after anthesis (DAA) in HOCL and LOCL, respectively, over three years. Furthermore, HOCL exhibited significantly higher fresh and dry matter at most developmental stages of siliques and seeds. From 20 DAA, lipids were deposited in the seed of HOCL significantly faster than that of LOCL, which was validated by transmission electron microscopy, showing that HOCL accumulates considerable more oil bodies in the seed cells. Markedly higher silique chlorophyll content was observed in HOCL consistently over the three consecutive years, implying a higher potential of photosynthetic capacity in siliques of HOCL. As a consequence, HOCL exhibited significantly higher content of fructose, glucose, sucrose, and starch mainly at 20 to 45 DAA, a key stage of seed lipid deposition. Moreover, seed sugar content was usually higher than silique indicating the importance of sugar transportation from siliques to seeds as substrate for lipid biosynthesis. The much lower silique cellulose content in HOCL was beneficial for lipid synthesis rather than consuming excessive carbohydrate for cell wall.

**Conclusions:**

Superior physiological characteristics of siliques in HOCL showed advantage to produce more photosynthetic assimilates, which were highly correlated to seed oil contents.

**Electronic supplementary material:**

The online version of this article (doi:10.1186/1999-3110-55-34) contains supplementary material, which is available to authorized users.

## Background

Canola (*Brassica napus* L.) seed oil content is an important and intricate quantitative trait, which is regulated by many factors such as genetic control, developmental cues, and environmental influences (Cheema et al. 2001; Delourme et al. 2006; Rathke et al. 2006; Wang et al. 2010). Proteins and carbohydrates are two closely-related major reserves in developing canola seed during lipid biosynthesis (Wang et al. 2007a; Ekman et al. 2008). Carbohydrate, in the form of photosynthetic assimilates from green tissues such as siliques and leaves, plays multiple roles in cellular metabolism. Firstly, carbohydrate such as sucrose produced by photosynthesis in green tissues is a precursor and essential substrate for fatty acid biosynthesis (Focks and Benning 1998). Thus, it implies that ample carbohydrates in canola siliques and seeds might be beneficial to higher seed oil content. Secondly, conversion of carbohydrate into lipids, proteins, and secondary metabolites such as fiber is an elaborate regulation system during seed development, which can greatly affect seed oil content (Ekman et al. 2008).

Lipid biosynthetic pathway at seed filling stage has been revealed in some plant species such as *Arabidopsis thaliana*, *Glycine max* L., and *Brassica napus* L. (Ruuska et al. 2002; Houston et al. 2009). As the starting point, sucrose is cleaved into fructose and glucose that take part in fatty acid metabolism (Baud and Lepiniec 2009; Hajduch et al. 2010). Thus, it emphasizes the essential role of those carbohydrates on the lipid biosynthesis in seeds of oil crops. Further, the crucial role of carbohydrate metabolism for seed development of oil crops such as soybean and canola was recently demonstrated by RNA transcript profiling, proteomic analysis, and functional studies of relevant genes (Hajduch et al. 2006; Andre et al. 2007; Agrawal et al. 2008; Houston et al. 2009).

In canola, the reproductive growth upon flower initiation becomes more dominant despite the vegetative growth is continuing. Its notable characteristics are the formation of more siliques and the senescence of old leaves at the lower stalk. At the end of flowering, siliques dominate the canopy of canola plants and become the major photosynthetic organ regardless of the existence of other green tissues (e.g. newly formed leaves at the base of branches and stems) (Berry and Spink 2006). Almost 60% of assimilates for seed development were derived from photosynthesis of siliques confirms the predominance of silique as a photosynthetic organ for seed development (Lewis and Thruling 1994; Gammelvind et al. 1996). Furthermore, the developing seed is directly connected with silique via funiculus (Stadler et al. 2005), hence silique might be the only organ that directly supplies marco- and micro-nutrients to the developing seed. Consequently, it is worthwhile to investigate the physiological roles of silique in seed oil accumulation. During last few decades, lots of efforts were elaborated on silique dehiscence (or pod shattering), which was directly related to seed disperses and yields (Jenkins et al. 1996; Child et al. 2003; Bennett et al. 2011). However, little work was conducted on the dynamic association between carbohydrates, chlorophyll content, and seed oil accumulation in siliques over long period of growth and across several seasons. Questions such as, whether the amount and composition of assimilates produced by silique can affect seed lipid accumulation, and whether the dynamic changes of assimilates correlate to the lipid content, biomass, and yield, still need to be addressed.

Previously, we employed a high oil content line (HOCL; 50.4% of oil content) and a low oil content line (LOCL; 41.4% of oil content) to evaluate the difference of macro-nutrients and carbohydrate content in above-ground tissues: flower, stem, and leaf. The result suggested that higher carbohydrate content and lower N content in those photosynthetic tissues might contribute to higher oil content in HOCL (Hua et al. 2012a). In the present study, dynamic changes of silique and seed weight of HOCL and LOCL were recorded to evaluate their growth characteristics. Seed oil content along with seed and silique ultrastructure of HOCL and LOCL were analyzed to elucidate the difference of seed oil deposition during seed development. Furthermore, chlorophyll content fructose, glucose, sucrose, starch, and cellulose were determined in both siliques and seeds of HOCL and LOCL. The correlation and factor analysis between seed oil content and these physiological indexes revealed the key roles of increasing silique chlorophyll content and seed starch content and reducing cellulose content during seed oil accumulation.

## Methods

### Plant materials

Field trials were conducted over three growth seasons in 2009–2010, 2010–2011, and 2011–2012, at the experimental station of Zhejiang Academy of Agricultural Sciences, Hangzhou, China. Seeds of two Recombinant Inbred Lines (RILs) with high and low oil content, developed from Zheshuang 6/Huyou 15, were sown in a seedling bed on October 3, 2009 and October 5, 2010 and 2011. After one month, seedlings with uniform growth were selected and transplanted to experimental plots and general field managements were conducted for all three trials. The soil type in the experimental station was loamy clay (loamy, mixed, and thermic Aeric Endoaquepts). Urea was evenly broadcasted in the soil at 150 kg ha^-1^ before transplanting and an additional 75 kg ha^-1^ urea was applied at the end of January for each season. During the three growth seasons, rainfall (1041.7, 1048.3, and 1217.5 mm, respectively) in Hangzhou was sufficient for canola without irrigation.

### Experimental design and sampling

The experiments employed a randomly complete block design, where two canola lines with contrasting seed oil content were used as two experimental treatments. Plants were grown in 20 × 3.2 m plots with three replications for both HOCL and LOCL. Each plot consisted of eight rows with 0.35 m between rows and 0.20 m between plants. Plants were tied with colored cotton strings at main inflorescence and the data were recorded from 10 days after anthesis (DAA) till 65 DAA. Five plants were randomly sampled in the core area of each plot to minimize marginal effect.

### Oil content measurement

The measurement of oil content was performed using a Shimadzu gas chromatography system (GC-2014, Japan) equipped with a flame ionization detector and a 30 m (length) × 0.25 mm (inner diameter) × 0.25 μm (liquid membrane thickness) column (Supelco wax-10, Supelco) in 2010 and 2011 using fresh immature seed samples. They were frozen in liquid nitrogen, ground into fine powder. The extraction of fatty acids and conditions of gas chromatography using heptadecanoate as an internal standard were essentially as described by Hajduch et al. (2006) and Mu et al. (2008). Briefly, samples were initially extracted by 1 mL of 14% boron trifluoride with heptadecanoate as standard in toluene. 150 μL standard in toluene and samples were incubated at 95°C for 90 min and shaking acutely with every 10 min. After incubation and cooled to room temperature, samples were added into 1 mL of water and 3 mL of hexane. Tubes were shaken vigorously and centrifuged at 4000 rpm for 5 min. Top phase was transferred into another new tube and re-extracted with 3 mL of hexane and 3 mL of 0.9% NaCl solution. The organic phase (acyl methyl estered fatty acids) was used for GC analysis. Soxhlet-based extraction method was applied to determine seed oil content with petroleum ether as the organic solvent in 2012 based on a seed dry weight. Briefly, seeds were ground into powder by a pulverizer (Wenlin Dalin Machine Com. Ltd, China). Five to six grams of sample were dried at 100°C for 4 h. Weight of dried samples were recorded and extracted by ethyl ether absolute for 24 h with a reflux condensation device. A low boiling point (lower than 60°C) extraction solvent ethyl ether was used to avoid direct extraction of membrane-bound lipids. The crude lipids were composed of free lipids in the seed. For the experiments in 2010 and 2011, fatty acids were used to stand for “seed oil content”. It was previously reported that there was strong correlation between fatty acids and seed oil content in the model plant *Arabidopsis*- a close relative to *Brassica napus* L. (Hobbs et al. 2004). In 2012, seed oil content was defined as crude lipids from canola seeds.

### Transmission electron microscopy (TEM)

Siliques and seeds were sampled from the main inflorescence of HOCL and LOCL of plants at 30, 40, and 50 DAA. In order to minimize sampling difference, the samples were taken at the same position of the main inflorescence of plants with very similar growth and height. Siliques of the two lines were cut into 0.5 × 0.5 cm small pieces and the middle portions were used. Seeds of the two lines were evenly cut into two parts along the axis of cotyledons. The procedure for the TEM technique was the same to the description by Chen et al. (2012). Briefly, the samples were fixed with 2.5% gluteraldehyde in sodium phosphate buffer, postfixed in osmium tetroxide, dehydrated, and embedded in Spurr’s resin. Transverse ultrathin sections were observed under a TEM (JEM-1230, JEOL, Japan).

### Silique fresh and dry weight and chlorophyll determination

Immature seeds were carefully detached from developing siliques and both were weighed immediately for fresh weight in 2010 and 2011. Since fresh weight-based carbohydrate measurement is usually affected by variable high water content in seeds, an absolute content of carbohydrate was also performed as on a seed dry weight basis. Therefore, in 2012, immature separated seeds and siliques were heated at 105°C for 30 min and were then dried at 70°C to a constant dry weight. Separated siliques in the consecutive three years were ground into fine powder with liquid nitrogen and extracted by 800 mL L^-1^ of acetone. The total chlorophyll content was estimated as described by Lichtenthaler (1987).

### Carbohydrate content measurement

To determine the dynamic changes of carbohydrates, fresh immature siliques and seeds from 2010 and 2011 seasons were ground into powder in liquid nitrogen while dried immature siliques and seeds were ground into powder by a pulverizer. About 100 mg powder was boiled twice in 10 mL of 800 mL L^-1^ ethanol for 30 min and centrifuged at 10000× *g* after the extract was cooled down to room temperature. One hundred milligrams of activated charcoal was added to the supernatant to remove chlorophyll. The chlorophyll-free supernatant was then used for determination of glucose, fructose, and sucrose according to Hendrix (1993). The starch in the pellets was digested with amyloglucosidase for 100 min at 55°C and determined according to Hendrix (1993). The remaining sediment was used to estimate cellulose content. The pellets were digested in an acetic-nitric reagent and the cellulose content was determined by anthrone according to the method described by Updegraff (1969).

### Statistical analysis

Means of all the physiological traits of two lines were compared using Duncan’s test at P < 0.05 at each developmental stage in 2010, 2011, and 2012. Correlation analysis between fatty acid content (lipid content) and each physiological indexes, namely, silique and seed weight, silique chlorophyll content, silique and seed fructose content, silique and seed glucose content, silique and seed sucrose content, silique and seed starch content, and silique and seed cellulose content, respectively, was performed during whole seed development. Factor analysis was also performed using these physiological parameters for dimension reduction. These analyses were performed using SPSS software.

## Results

### Dynamics of fresh and dry weight in developing silique and seed of HOCL and LOCL

#### Silique fresh and dry weight

**Figure 1:**
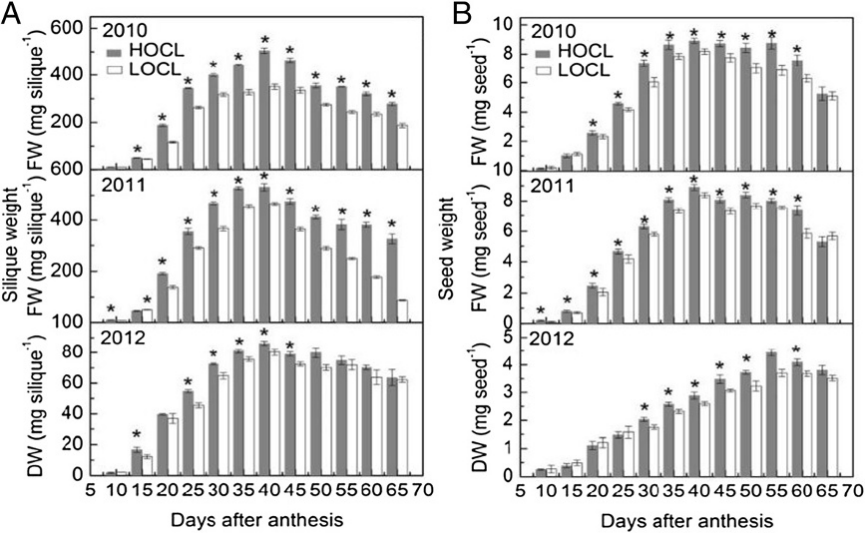
**Dynamic of silique fresh weight in 2010, 2011, and dry weight in 2012 (A) and seed fresh weight in 2010, 2011, and dry weight in 2012 (B) of canola high oil content line (HOCL) and low oil content line (LOCL) from 10 to 65 days after anthesis (DAA) at a 5-day interval.** Each value is the mean of 15 main-inflorescence siliques of five plants from each of three replicate plots. Error bars represent standard errors. “*” at each developmental stage indicates a significant difference at 5% probability between HOCL and LOCL in both years.

After fertilization, siliques developed very quickly from 10 to 30 DAA in both canola lines with a near-linear increment in each year (Figure 1A). Around 79% and 88% in HOCL and 91% and 79% in LOCL of silique fresh matter was produced before 30 DAA in 2010 and 2011, respectively. From 10 to 30 DAA, silique fresh weight increased with an average of 19.6 and 22.9 mg day^-1^ in HOCL in 2010 and 2011, respectively, which were much higher than those in LOCL (15.4 and 17.9 mg day^-1^). From 30 to 40 DAA, growth rate of silique in both lines was much smaller than that of the previous stages. There was significant difference of silique fresh weight between two lines. Notably, compared with LOCL, HOCL produced more fresh matters than the LOCL from 20 DAA onwards, yielding more fresh matter by 44% and 14% at 40 DAA in 2010 and 2011, respectively. Silique dry weight also accumulated with high speed and produced 84% and 89% of total dry matter before 30 DAA in both lines, which indicated an average of 2.4 and 2.1 mg day^-1^ of growth rate in HOCL and LOCL (Figure 1A). Decrease of silique dry weight occurred from 40 DAA in both HOCL and LOCL. Significantly higher silique dry weight was found in HOCL mainly from 15 to 50 DAA with an increment up to 9.8 mg day^-1^ of growth rate as a comparison with LOCL (Figure 1A).

#### Seed fresh and dry weight

Generally, similar near-linear increments of fresh and dry weight in both HOCL and LOCL were observed in developing seeds in comparison to developing siliques from 10 to 30 DAA, but this trend was extended to 40 DAA in 2010 and 2011, and 55 DAA in 2012, respectively (Figure 1B). The average seed growth rate reached 0.29 mg day^-1^ in HOCL for both years and at 0.25 and 0.27 mg day^-1^ in LOCL in 2010 and 2011, respectively. Seed fresh weight then underwent a steady period with a relatively higher and stable weight for 2 weeks in HOCL and LOCL from 40 to 55 DAA in two years (Figure 1B) and, thereafter, decreased due to dehydration at seed maturation stage. Marked difference of seed fresh weight between HOCL and LOCL was found from 20 to 55 DAA. Although seed fresh weight peaked at 40 DAA for both HOCL and LOCL, the biggest gap of seed fresh weight was at 55 and 60 DAA, which showed a faster decrease of 22% and 21% in LOCL in comparison with HOCL in 2010 and 2011 (Figure 1B). Different from seed fresh weight, seed dry weight kept increasing from 10 to 55 DAA in both lines and reduced from 55 DAA, which was much more in accordance with seed fresh weight in 2010 and 2011 (Figure 1B). Seed dry weight of LOCL was 83.3% of HOCL at 55 DAA (Figure 1B). Therefore, seed physiological maturation commenced from 55 DAA for both HOCL and LOCL (Figure 1B).

Taken together, HOCL exhibited significantly higher silique and seed fresh and dry weight, indicating a higher potential to produce higher seed oil content.

### Dynamic of seed oil content in HOCL and LOCL

**Figure 2:**
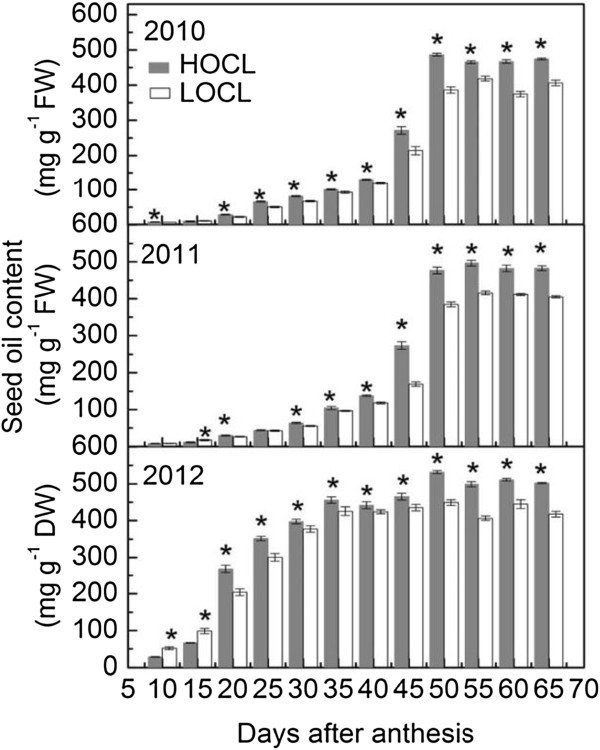
**Dynamic of seed oil content based on fresh weight in 2010, 2011, and that on dry weight in 2012 in canola high oil content line (HOCL) and low oil content line (LOCL) from 10 to 65 days after anthesis (DAA) at a 5-day interval.** Each value is the mean of 15 siliques from main inflorescence, five from each of three replicate plots in 2010 and 2011. Bars in the open and solid squares are standard errors. “*” at each developmental stage indicates a significant difference at 5% probability between HOCL and LOCL in both years.

**Figure 3:**
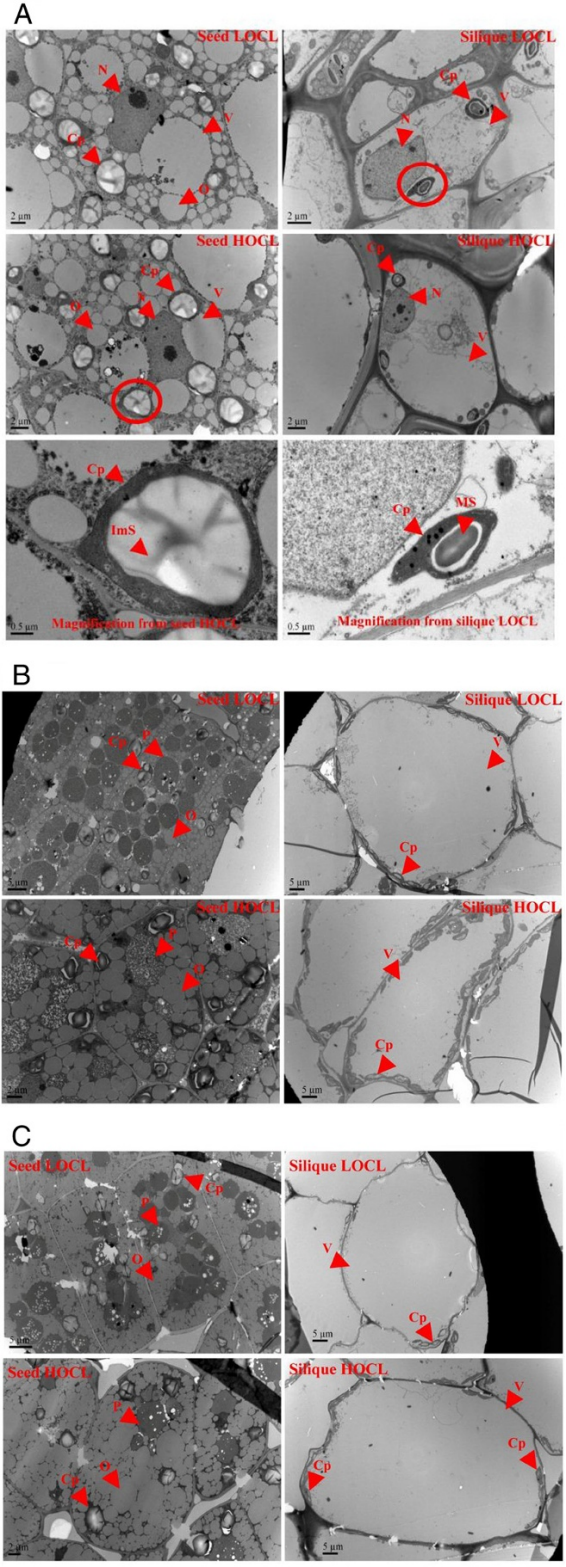
**Ultrastructure of silique (right panel) and seed (left panel) at 30 (A), 40 (B), and 50 (C) DAA by transmission electron microscopy (TEM).** The circle in **(A)** is a magnification of chloroplast in the seed and silique. Cp, chloroplast; ImS, immature starch grain; MS, matured starch grain; N, nucleolus; O, oil body; P, protein; V, vacuole. Images are one representative out of 10 captured in 2012.

**Figure 4:**
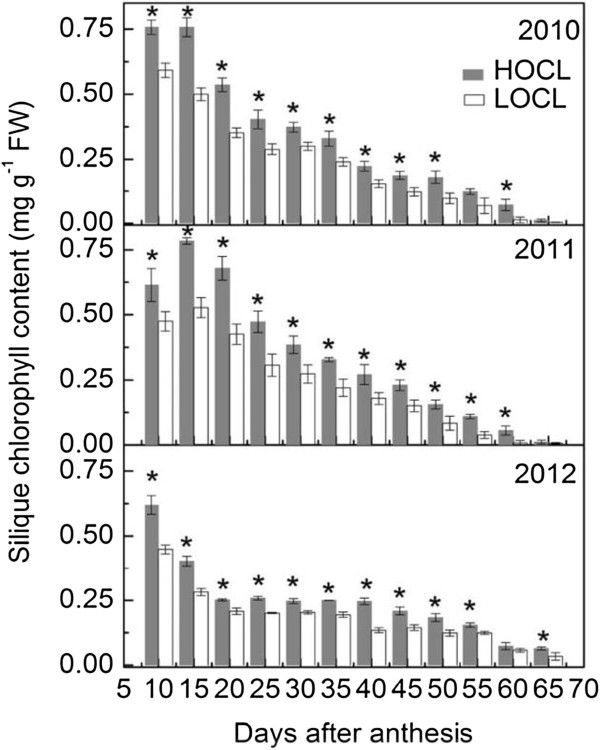
**Dynamic chlorophyll content in developing silique of canola high oil content line (HOCL) and low oil content line (LOCL) from 10 to 65 days after anthesis (DAA) at a 5-day interval in 2010, 2011, and 2012.** Each value is the mean of 15 main-inflorescence siliques of five plants from each of three replicate plots. Error bars represent standard errors. “*” at each developmental stage indicates a significant difference at 5% probability between HOCL and LOCL in both years.

**Figure 5:**
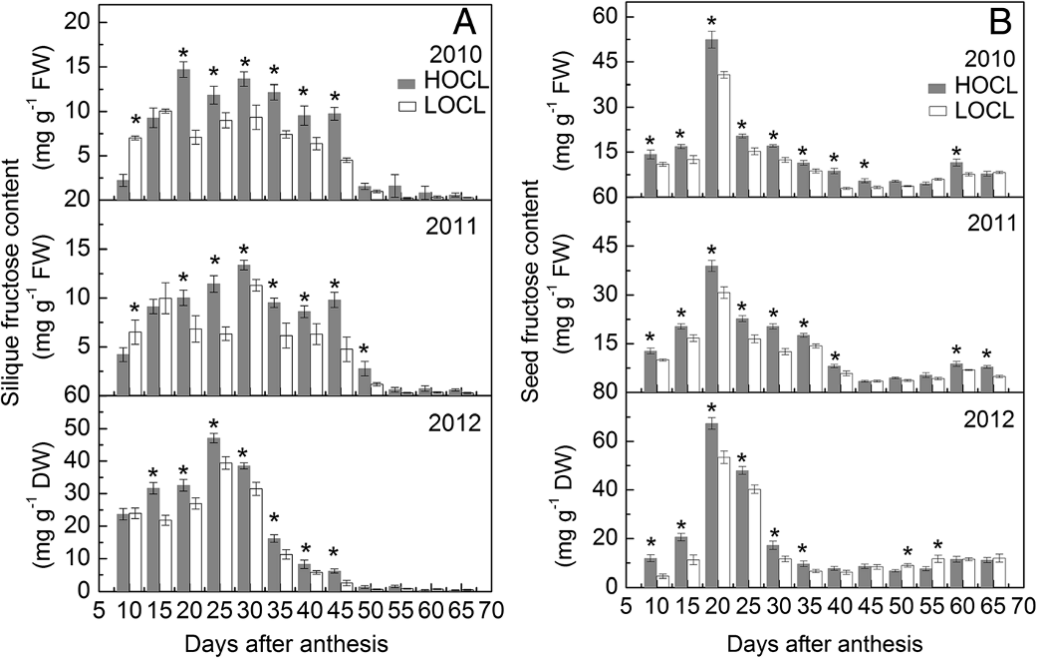
**Dynamic of silique fructose content based on fresh weight in 2010, 2011, and that on dry weight in 2012 (A) and seed fructose content based on fresh weight in 2010, 2011, and that on dry weight in 2012 (B) of canola high oil content line (HOCL) and low oil content line (LOCL) from 10 to 65 days after anthesis (DAA) at a 5-day interval.** Each value is the mean of 15 siliques from main inflorescence, five from each of three replicate plots. Bars in the open and solid squares are standard errors. “*” at each developmental stage indicates a significant difference at 5% probability between HOCL and LOCL in both years.

**Figure 6:**
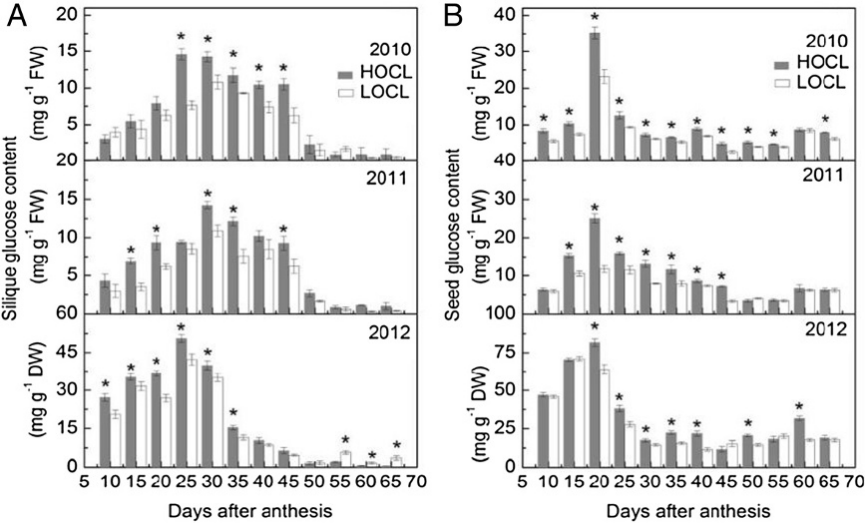
**Dynamic of silique glucose content based on fresh weight in 2010, 2011, and that on dry weight in 2012 (A) and seed glucose content based on fresh weight in 2010, 2011, and that on dry weight in 2012 (B) of canola high oil content line (HOCL) and low oil content line (LOCL) from 10 to 65 days after anthesis (DAA) at a 5-day interval.** Each value is the mean of 15 siliques from main inflorescence, five from each of three replicate plots. Bars in the open and solid squares are standard errors. “*” at each developmental stage indicates a significant difference at 5% probability between HOCL and LOCL in both years.

**Figure 7:**
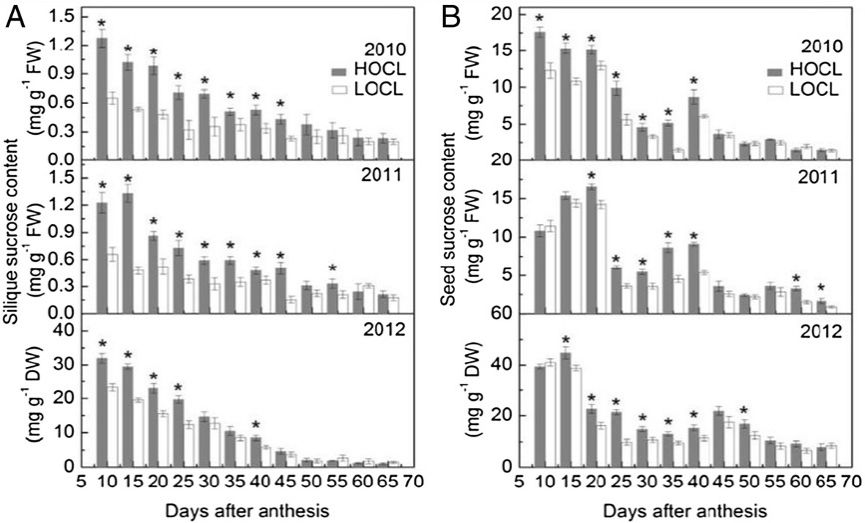
**Dynamic of silique sucrose content based on fresh weight in 2010, 2011, and that on dry weight in 2012 (A) and seed sucrose content based on fresh weight in 2010, 2011, and that on dry weight in 2012 (B) of canola high oil content line (HOCL) and low oil content line (LOCL) from 10 to 65 days after anthesis (DAA) at a 5-day interval.** Each value is the mean of 15 siliques from main inflorescence, five from each of three replicate plots. Bars in the open and solid squares are standard errors. “*” at each developmental stage indicates a significant difference at 5% probability between HOCL and LOCL in both years.

Seed oil content in the three consecutive growth seasons was monitored from 10 to 60 DAA (Figure 2). Before 30 DAA, oil content was only a small proportion of seed fresh weight in both lines indicating other metabolites such as carbohydrates (Figures 3, 4, 5, 6, 7) but not lipids depositing in the liquid endosperm in 2010 and 2011. Along with the increasing seed weight (Figure 1B), oil content rose dramatically before 55 DAA in 2010 and 2011 (Figure 2). Although the difference of oil content between HOCL and LOCL at early seed developing stage was statistically significant, this gap of oil content became significantly larger after 45 DAA in 2010 and 2011 (Figure 2). Compared to LOCL, the maximum oil content of HOCL were 16% and 19% higher in 2010 and 2011, respectively (Figure 2). Similarly, lipids content on a dry weight basis accumulated in developing seed with high speed from 20 to 50 DAA without much difference between two lines. HOCL showed constantly 16% more lipid content than LOCL after 50 DAA in a dry weight basis. A common property worth noting in fatty acid/lipids content during seed development was that the highest amount was not at the full maturation stage at 65 DAA but at the beginning of seed dehydration at 50 DAA (Figure 2).

### TEM of silique and seed in HOCL and LOCL

The significant difference between HOCL and LOCL of seed oil content during seed development led us to compare their silique and seed ultrastructure at 30, 40, and 50 DAA (Figure 3) representing three seed oil formation stages, namely, early, middle, and late stages, respectively. At 30 DAA, seed cells were mainly composed of nucleolus, large vacuoles, chloroplasts, and oil bodies. Chloroplast was almost round in shape and filled with developing starch granule. HOCL contained more numbers of chloroplasts (6.7 per cell) than that of LOCL (5.5 per cell) (Figure 3A). Different from seed cell, silique cell mainly consisted of nucleolus, vacuoles, and chloroplasts without oil bodies. Furthermore, silique chloroplast appeared in oval shape and with relatively small proportion of matured starch granule (Figure 3A). At 40 DAA, large amount of proteins accumulated in seed cells of LOCL but oil bodies were more predominate in seed cells of HOCL. Furthermore, more chloroplasts were also found in HOCL (4.6 per cell) than that in LOCL (3.2 per cell). Unlike at previous stages, it was found that single silique cell was mainly occupied by a large vacuole merged from many small ones and chloroplasts were pushed near the cellular membrane. There was no difference between two lines except the number of chloroplast in silique cell (Figure 3B). Averagely, HOCL contained 17.6 chloroplasts per cell while LOCL contain 13.2 ones. Furthermore, starch grain in the chloroplast of seed cell matured (Figure 3B) at 50 DAA, a similar ultrastructure was observed in comparison with that at 40 DAA (Figure 3C). Thus, it was clear that 1.) seed cell in HOCL contained more chloroplasts, oil bodies and less proteins while the reverse result in LOCL; 2.) silique cell in HOCL had more chloroplasts than that in LOCL at developmental stages.

### Dynamic of silique chlorophyll content in HOCL and LOCL

Overall, chlorophyll content in silique kept on decreasing in both HOCL and LOCL in 2010, 2011, and 2012 (Figure 4). Silique chlorophyll content in HOCL was significantly higher than that in LOCL at most developmental stages. Silique chlorophyll contents in HOCL, averaged from 10 to 30 DAA, were 28% 31%, and 31% higher than those of LOCL in 2010, 2011, and 2012, respectively (Figure 4). Those results along with a higher abundance of chloroplasts in silique cells of HOCL lead to our assumption that a higher photosynthetic production of carbohydrates in HOCL.

### Dynamics of silique and seed carbohydrate content in HOCL and LOCL

#### Fructose content

Fructose belongs to hexose and is involved in many physiological metabolisms (Mccutchan and Monson 2001). Silique fructose content was significantly higher in HOCL than that of LOCL at 20 to 40 DAA while no difference after 40 DAA between two lines across three years (Figure 5A), indicating 20 to 40 DAA was the key stage for the differentiation of silique fructose metabolism between HOCL and LOCL. Silique fructose content in HOCL and LOCL declined to near zero level after 40 DAA suggesting that it was mostly converted in silique or transported into seed. Silique fructose content of HOCL were, in average, 48% and 57% higher than that of LOCL in 2010 and 2011 based on fresh weight and was 48% higher than that of LCOL in 2012 on a dry weight basis from 20 to 40 DAA (Figure 5A).

Fructose content in developing seeds illustrated a very similar kinetic trend in both lines from 2010 to 2012 (Figure 5B). HOCL and LOCL showed a large increase of fructose content, peaked at 20 DAA and then dropped rapidly close to zero. For the three years, peak seed fructose content in LOCL was only 77.5%, 78.8%, and 79.3% of those of HOCL. However, there was little difference in seed fructose content between two lines after 40 DAA over three years.

#### Glucose content

Similar to the trend of fructose content in developing siliques was observed for glucose content (Figure 6A). From 20 to 45 DAA, HOCL showed significantly higher glucose content than that of LOCL. The maximum glucose content in developing siliques of HOCL was found at 25 DAA of 2010 and 2012 and at 30 DAA of 2011, which synthesized 47.5%, 16.5%, and 23.4% more silique glucose as compared with LOCL. The glucose content dropped to very low level from 50 DAA indicating an active stage from 20 to 45 DAA of glucose metabolism (Figure 6A).

In general, glucose content in developing seed varied similar to the seed fructose content (Figures 5B, 6B). We found that seed glucose content accumulated rapidly before 25 DAA and then declined dramatically in both lines in three years. Slightly different from developing silique, seed glucose content exhibited a small recovery in two lines during seed maturation, which was especially obvious for HOCL at 60 DAA in 2012 (Figure 6B). Significant difference of glucose content between HOCL and LOCL mostly found from 15 to 45 DAA. HOCL reached its peak abundance of glucose content at 20 DAA over the three consecutive years, when seed glucose content in LOCL were only 65.9%, 46.5%, and 78.1% of that in HOCL, respectively (Figure 6B).

#### Sucrose content

Sucrose is an essential photo-assimilate, cleaved into fructose and glucose by invertase and sucrose synthase (Fallahi et al. 2008). Overall, sucrose content in silique was constantly in decline over time in both HOCL and LOCL in three years (Figure 7A). The large difference of sucrose content in silique was at 10 to 30 DAA between two lines regardless of the fresh or dry basis, indicating most of the sillque sucrose was possibly cleaved during early flowering stage but not late flowering or seed filling stage (Figure 7A).

Twenty DAA was the turning point where higher seed sucrose content was found in both lines in 2010 and 2011 and it then reduced to relatively lower values. Interestingly, seed sucrose content exhibited a second peak around 35 to 45 DAA in two lines during seed maturation of each year (Figure 7B). The remarkable distinction of seed sucrose content in two lines was also mainly observed from 20 to 45 DAA. Similarly, high seed sucrose content at early stage (10 to 15 DAA) was also detected compared to the peaks of fructose and glucose in seeds (Figure 7B). On average, seed sucrose content in HOCL were 83%, 60%, and 54% more abundant in comparison to that in LOCL from 20 to 40 DAA in 2010, 2011, and 2012, respectively (Figure 7B).

#### Starch content

**Figure 8:**
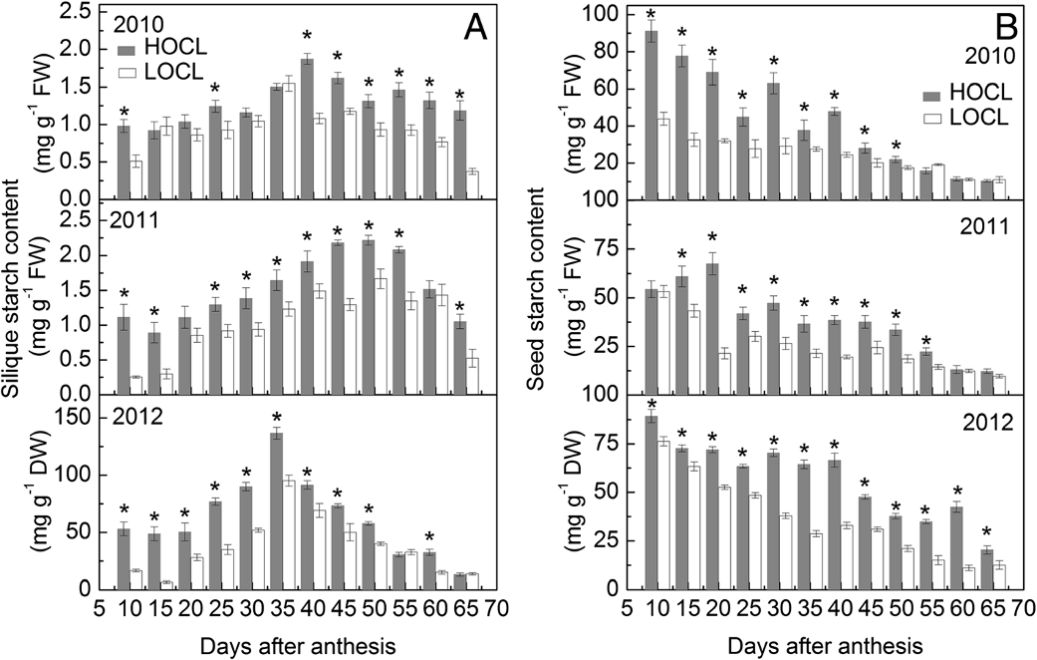
**Dynamic of starch content based on fresh weight in 2010, 2011, and that on dry weight in 2012 (A) and seed starch content based on fresh weight in 2010, 2011, and that on 2012 (B) of canola high oil content line (HOCL) and low oil content line (LOCL) from 10 to 65 days after anthesis (DAA) at a 5-day interval.** Each value is the mean of 15 siliques from main inflorescence, five from each of three replicate plots. Bars in the open and solid squares are standard errors. “*” at each developmental stage indicates a significant difference at 5% probability between HOCL and LOCL in both years.

Starch synthesized in both siliques and seeds is considered as one of the major carbon suppliers for fatty acid synthesis (Andriotis et al. 2010). Thus, we hypothesized that silique and seed starch content in HOCL is higher than those in LOCL. Different from the dynamic changes of silique glucose, fructose and sucrose content, starch content accumulated from early flowering stage, peaked at 35 to 45 DAA and decreased gradually over time in HOCL and LOCL across three seasons (Figure 8A). The silique starch content varied across three seasons. HOCL only showed significantly and consistently higher starch content from 40 DAA in 2010, but exhibited significantly higher silique starch content than that of LOCL across the whole 65 day period in both 2011 and 2012. For instance, in 2012, the silique starch content in HOCL and LOCL were both peaked at 35 DAA, but HOCL accumulated a 30.3% more starch content compared to LOCL (Figure 8A).

Seed starch content in two lines was much higher based on fresh weight than silique starch content (Figure 8A,B). Furthermore, seed starch content in both lines generally declined from 10 DAA onwards across three seasons with some fluctuations. For example, there was a short period of increase in seed starch content from 10 to 20 DAA in HOCL in 2011 (Figure 8B). Figure 8B also illustrated that starch content in developing seed in HOCL was significant higher than that in LOCL in the majority of developmental stages. Across the 12 measured seed developmental stages, average starch content in HOCL increased 43%, 37%, and 37% more than that of LOCL in the consecutive three years (Figure 8B).

#### Cellulose content

**Figure 9:**
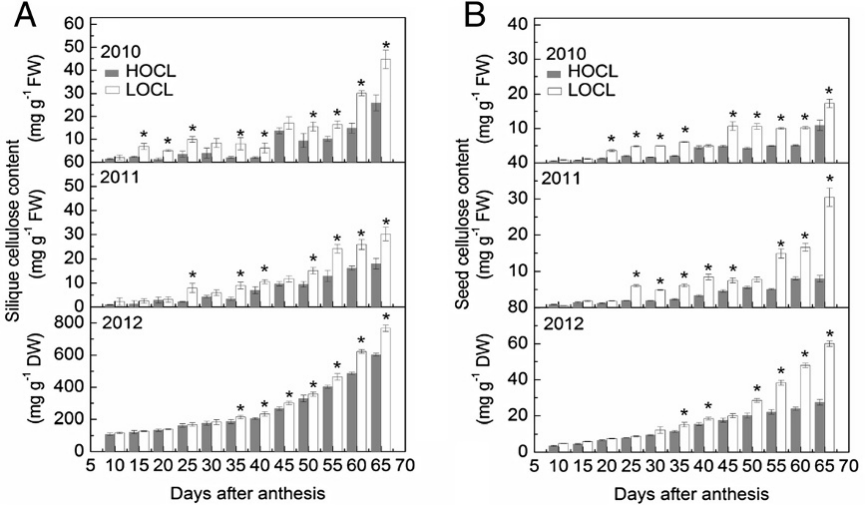
**Dynamic of cellulose content based on fresh weight in 2010, 2011, and that on dry weight in 2012 (A) and seed cellulose content based on fresh weight in 2010, 2011, and that on dry weight in 2012 (B) of canola high oil content line (HOCL) and low oil content line (LOCL) from 10 to 65 days after anthesis (DAA) at a 5-day interval.** Each value is the mean of 15 siliques from main inflorescence, five from each of three replicate plots. Bars in the open and solid squares are standard errors. “*” at each developmental stage indicates a significant difference at 5% probability between HOCL and LOCL in both years.

Cellulose is the major component of plant cell wall, however, too much cellulose synthesized in siliques and seeds would consume excessive carbohydrates, which could have been used for synthesis of fatty acids (Louvet et al. 2011). Therefore, our hypothesis was that HOCL should synthesize less cellulose content than LOCL. We observed an overall increase of silique cellulose content across the growth stages, both in HOCL and LOCL, which was more pronounced from 55 DAA over three years (Figure 9A). However, HOCL showed distinctively less cellulose content than that in HOCL at the end of silique development. The largest difference of cellulose content between HOCL and LOCL was found at 65 DAA reaching 18.9 and 12.2 mg g^-1^ fresh weight in 2010 and 2011, respectively (Figure 9A). However, dry-weight based silique cellulose content of HOCL was significantly lower (21%) than that of LOCL at 65 DAA (Figure 9A).

Similar deposition dynamics of cellulose in developing seed was observed in HOCL and LOCL over three years (Figure 9B). Significantly lower seed cellulose content in HOCL was found in contrast to LOCL from 35 to 65 DAA over three years. For instance, there was 37%, 74%, and 54% less seed cellulose content in HOCL in comparison with LCOL at 65 DAA in 2010, 2011, and 2012, respectively (Figure 9B).

### Correlation between seed oil content and physiological indexes and factor analysis

The above results demonstrated that significant difference of physiological indexes exists between HOCL and LOCL during lipid biosynthesis. In order to understand how these factors were related and contributed to seed oil content during seed development, a correlation analysis between seed oil content and physiological indexes and factor analysis were performed using these physiological indexes. Tables 1, 2, and 3 showed that lipids were positively correlated with silique and seed weight except silique weight in LOCL in 2011. Seed lipids were very significantly associated with silique total chlorophyll in both lines under three experimental years. This indicated that decreasing silique chlorophyll content during seed development does not benefit seed oil accumulation. As for sugars, it was found that silique fructose, glucose, sucrose, and seed starch content were significantly and negatively correlated with lipids in both lines from 2010 to 2012. Generally, seed fructose, glucose, sucrose, and silique starch content showed similar correlations although some are not significant. This revealed that these physiological parameters were more environmentally dependent over three years. It was observed that silique and seed cellulose content was significantly related with lipids. The result illustrated that the more cellulose synthesized, the more adverse effect on seed lipids.

**Table 1:** **Correlation among the physiological traits and seed oil content in 2010 at seed developmental stages: the value in the upper triangle is the Person correlation coefficient in canola high oil content line (HOCL) while the lower triangle is that in canola low oil content line (LOCL)**

Parameters	Seed oil content	Silique weight	Seed weight	Total chlorophyll	Silique fructose	Seed fructose	Silique glucose	Seed glucose	Silique sucrose	Seed sucrose	Silique starch	Seed starch	Silique cellulose	Seed cellulose
Seed oil content	1	0.30	0.53	-0.86**	-0.96**	-0.48	-0.74**	-0.38	-0.80**	-0.72**	-0.27	-0.88**	0.79**	0.88**
Silique weight	0.33	1	0.94**	-0.65**	-0.16	-0.44	0.36	-0.35	-0.71**	-0.75**	0.60**	-0.51	0.12	0.39
Seed weight	0.56	0.93**	1	-0.79**	-0.42	-0.55	0.10	-0.43	-0.78**	-0.83**	0.53	-0.66**	0.28	0.55
Total chlorophyll	-0.85**	-0.72**	-0.81	1	0.79**	0.42	0.41	0.26	0.96**	0.83**	-0.01	0.96**	-0.76**	-0.88**
Silique fructose	-0.75**	0.20	-0.06	0.40	1	0.38	0.81**	0.26	0.68**	0.57	0.37	0.79**	-0.75**	-0.83**
Seed fructose	-0.55	-0.37	-0.53	0.49	0.58*	1	0.22	0.94**	0.45	0.64**	-0.12	0.41	-0.30	-0.40
Silique glucose	-0.65**	0.43	0.13	0.22	0.89**	0.25	1	0.13	0.30	0.15	0.65**	0.53	-0.65**	-0.55
Seed glucose	-0.42	-0.33	-0.47	0.37	0.47	0.97**	0.13	1	0.31	0.58*	-0.14	0.25	-0.21	-0.32
Silique sucrose	-0.85**	-0.71**	-0.83**	0.97**	0.39	0.56	0.22	0.45	1	0.87**	-0.06	0.94**	-0.72**	-0.86**
Seed sucrose	-0.82**	-0.71**	-0.84**	0.93**	0.36	0.59*	0.16	0.54	0.95**	1	-0.23	0.79**	-0.61**	-0.76**
Silique starch	0.30	0.86**	0.83**	-0.62**	0.02	-0.48	0.23	-0.38	-0.60**	-0.51	1	0.08	-0.47	-0.25
Seed starch	-0.90**	-0.60**	-0.73**	0.95**	0.48	0.52	0.33	0.39	0.97**	0.91**	-0.51	1	-0.84**	-0.90**
Silique cellulose	0.81**	0.17	0.29	-0.74**	-0.62**	-0.44	-0.53	-0.33	-0.72**	-0.70**	0.10	-0.77**	1	0.90**
Seed cellulose	0.79**	0.35	0.42	-0.83**	-0.57	-0.47	-0.45	-0.31	-0.78**	-0.70**	0.34	-0.80**	0.23	1

*, **indicate two parameters are significant correlated at P < 0.05 and P < 0.01, respectively.

**Table 2:** **Correlation among the physiological traits and seed oil content in 2011 at seed developmental stages: the value in the upper triangle is the Person correlation coefficient in canola high oil content line (HOCL) while the lower triangle is that in canola low oil content line (LOCL)**

Parameters	Seed oil content	Silique weight	Seed weight	Total chlorophyll	Silique fructose	Seed fructose	Silique glucose	Seed glucose	Silique sucrose	Seed sucrose	Silique starch	Seed starch	Silique cellulose	Seed cellulose
Seed oil content	1	-0.01	0.57	-0.88**	-0.91**	-0.66**	-0.73**	-0.67**	-0.73**	-0.69**	0.49	-0.73**	0.94**	0.80**
Silique weight	0.35	1	0.80**	-0.35	0.10	-0.22	0.61**	-0.15	-0.44	-0.50	0.73**	-0.39	-0.02	-0.08
Seed weight	0.58*	0.94	1	-0.82**	-0.45	-0.60**	0.07	-0.55	-0.81**	-0.81**	0.86**	-0.76**	0.54	0.43
Total chlorophyll	-0.88**	-0.67	-0.79**	1	0.81**	0.70**	0.39	0.66**	0.85**	0.89**	-0.67**	0.85**	-0.90**	-0.80**
Silique fructose	-0.81**	0.13	-0.14	0.60**	1	0.56	0.77**	0.61**	0.57	0.59*	-0.43	0.64**	-0.87**	-0.73**
Seed fructose	-0.70**	-0.36	-0.56	0.73**	0.58*	1	0.39	0.87**	0.63**	0.72**	-0.40	0.28	-0.62**	-0.52
Silique glucose	-0.75**	0.30	0.02	0.46	0.95**	0.50	1	0.48	0.20	0.11	0.04	0.20	-0.65**	-0.54
Seed glucose	-0.70**	-0.27	-0.46	0.72**	0.69**	0.96**	0.58*	1	0.58*	0.58*	-0.44	0.29	-0.55	-0.39
Silique sucrose	-0.82**	-0.77	-0.85**	0.95**	0.44	0.54	0.31	0.51	1	0.84**	-0.59*	0.76**	-0.72**	-0.66**
Seed sucrose	-0.78**	-059*	-0.68**	0.90**	0.47	0.75**	0.39	0.74**	0.82**	1	-0.61**	0.68**	-0.73**	-0.66**
Silique starch	0.51	0.69**	0.84**	-0.59*	-0.16	-0.63**	-0.06	-0.57	-0.60**	-0.55	1	-0.66**	0.36	0.13
Seed starch	-0.86**	-0.51	-0.64**	0.94**	0.68**	0.71**	0.57*	0.72**	0.85**	0.86**	-0.39	1	-0.77**	-0.71
Silique cellulose	0.93**	0.37	0.53	-0.89**	-0.74**	-0.63**	-0.67**	-0.60**	-0.83**	-0.75**	0.337	-0.89**	1	0.94
Seed cellulose	0.93**	0.37	0.54	-0.89**	-0.74**	-0.66**	-0.68**	-0.63**	-0.82**	-0.77**	0.34	-0.91**	0.94**	1

*, **indicate two parameters are significant correlated at P < 0.05 and P < 0.01, respectively.

**Table 3:** **Correlation among the physiological traits and seed oil content in 2012 at seed developmental stages: the value in the upper triangle is the Person correlation coefficient in canola high oil content line (HOCL) while the lower triangle is that in canola low oil content line (LOCL)**

Parameters	Seed oil content	Silique weight	Seed weight	Total chlorophyll	Silique fructose	Seed fructose	Silique glucose	Seed glucose	Silique sucrose	Seed sucrose	Silique starch	Seed starch	Silique cellulose	Seed cellulose
Seed oil content	1	0.97**	0.90**	-0.86**	-0.62**	-0.25	-0.60**	-0.88**	-0.93**	-0.56	0.50	-0.92**	0.58*	0.62**
Silique weight	0.94**	1	0.83**	-0.80**	-0.57	-0.22	-0.55	-0.85**	-0.87**	-0.65**	0.63	-0.85**	0.44	0.49
Seed weight	0.92**	0.81**	1	-0.89**	-0.81*	-0.28	-0.78**	-0.76**	-0.98**	-0.21	0.17	-0.97**	0.81**	0.84**
Total chlorophyll	-0.89**	-0.77**	-0.84**	1	0.62**	0.01	0.56	0.60**	0.92**	0.35	-0.11	0.92**	-0.77**	-0.79**
Silique fructose	-0.60**	-0.48	-0.81**	0.51	1	0.56	0.97**	0.50	0.79**	-0.19	-0.07	0.74**	-0.71**	-0.74**
Seed fructose	-0.32	-0.35	-0.52	0.10	0.68**	1	0.53	0.46	0.25	-0.31	-0.19	0.25	-0.24	-0.27
Silique glucose	-0.63**	-0.52	-0.83**	0.53	0.99**	0.70**	1	0.53	0.76**	-0.15	-0.11	0.71**	-0.68**	-0.71**
Seed glucose	-0.74**	-0.76**	-0.74**	0.47	0.54	0.72**	0.57*	1	0.77**	0.48	-0.53	0.75**	-0.479	-0.515
Silique sucrose	-0.95**	-0.86**	-0.98**	0.87**	0.78**	0.49	0.81**	0.76**	1	0.29	-0.22	0.96**	-0.77**	-0.80**
Seed sucrose	-0.77**	-0.87**	-0.54	0.56	0.15	0.04	0.19	0.58*	0.59*	1	-0.76**	0.276	0.19	0.16
Silique starch	0.04	0.31	-0.21	0.21	0.33	-0.03	0.29	-0.20	0.13	-0.52	1	-0.21	-0.28	-0.23
Seed starch	-0.78**	-0.58*	-0.88**	0.84**	0.72**	0.35	0.73**	0.55	0.87**	0.25	0.482	1	-0.81**	-0.84**
Silique cellulose	0.68**	0.44	0.82**	-0.77**	-0.75**	-0.40	-0.76**	-0.49	-0.80**	-0.12	-0.59*	-0.92**	1	0.99**
Seed cellulose	0.83**	0.66**	0.94**	-0.83**	-0.84**	-0.50	-0.86**	-0.65**	-0.94**	-0.33	-0.42	-0.94**	0.94**	1

*, **indicate two parameters are significant correlated at P < 0.05 and P < 0.01, respectively.

There were many physiological indexes and their contributions to seed oil accumulation may not be understood only based on the correlation analysis. We simplified the factors to reflect their contributions using factor analysis. All of these 13 physiological parameters could be explained by the first three factors, which were accounted for more than 90% of variance in both lines during three experimental seasons (Table 4). Thus, these three factors can be stand for the 14 physiological parameters when it came to the seed oil content during seed development.

**Table 4:** **Total variance explained for each factor based on 14 physiological indexes of canola high oil content line (HOCL) and low oil content line (LOCL) from 2010 to 2012**

Factors	HOCL 2010		LOCL 2010		HOCL 2011		LOCL 2011		HOCL 2012		LOCL 2012	
	% of variance	Cumulative %	% of variance	Cumulative %	% of variance	Cumulative %	% of variance	Cumulative %	% of variance	Cumulative %	% of variance	Cumulative %
1	60.3	60.3	59.5	59.5	67.4	67.4	62.2	62.2	66.4	66.4	63.7	63.7
2	21.5	81.8	22.9	82.4	18.9	86.3	20.5	82.7	17.7	84.1	19.9	83.6
3	10.3	92.1	10.4	92.8	7.5	93.8	8.3	91.0	10.5	94.6	10.5	94.1
4	3.7	95.8	3.4	96.2	3.2	97.0	4.9	95.9	2.9	97.5	3.3	97.4
5	2.7	98.5	1.5	97.7	1.2	98.2	1.6	97.5	1.1	98.6	1.1	98.5
6	0.5	99.0	1.1	98.8	1.0	99.2	1.0	98.5	0.6	99.2	0.9	99.4
7	0.4	99.4	0.6	99.4	0.4	99.6	0.7	99.2	0.5	99.7	0.4	99.8
8	0.3	99.7	0.4	99.8	0.2	99.8	0.5	99.7	0.2	99.9	0.1	99.9
9	0.1	99.8	0.1	99.9	0.1	99.9	0.2	99.9	0.1	100.0	0.1	100.0
10	0.2	100.0	0.1	100.0	0.1	100.0	0.1	100.0				
11												
12												
13												
14												

In order to further reveal the distribution of these physiological parameters in these three groups, a rotate component matrix was listed in the Table 5. Factor 1, which accounted for 59% ~67% of the variation in different years of both lines, was strongly associated with total silique chlorophyll content, seed starch content, and silique and seed cellulose content. Factor 2, which accounted for 17% ~ 22% of the variation in different years of both lines, was mainly associated with silique and seed weight. Factor 3, which accounted for 7.5% ~ 10.5% of the variation in different years of both lines, was mainly correlated with seed fructose and glucose content. They could be regarded as carbohydrate source and polysaccharide factor, weight factor and seed hexose factor.

**Table 5:** **Principal factor matrix after varimax rotation for 14 physiological factors of canola high oil content line (HOCL) and low oil content line (LOCL) canola from 2010 to 2012: numbers in bond are those with factors loading greater than 0.70 (absolute value)**

Physiological parameters	HOCL 2010			LOCL 2010			HOCL 2011			LOCL 2011			HOCL 2012			LOCL 2012		
	F1*	F2*	F3*	F1	F2	F3	F1	F2	F3	F1	F2	F3	F1	F2	F3	F1	F2	F3
Fatty acids	-0.90	0.27	-0.26	-0.92	-0.06	-0.29	-.085	0.34	-0.33	-0.86	0.16	-0.45	-0.55	-0.80	-0.24	-0.74	-0.64	-0.15
Silique weight	-0.05	**0.99**	-0.08	-0.33	**0.90**	-0.20	-0.02	**0.99**	-0.08	0.20	**0.97**	-0.02	-0.28	-0.91	-0.28	-0.64	-0.75	-0.14
Seed weight	-0.24	**0.91**	-0.24	-0.50	**0.78**	-0.30	-.019	**0.92**	-0.29	-0.34	**0.88**	-0.30	-0.69	-0.55	-0.45	-0.92	-0.29	-0.23
Total chlorophyll	**0.72**	-0.68	0.10	**0.92**	-0.36	0.12	**0.70**	-0.64	0.30	**0.76**	-0.52	0.38	**0.76**	0.62	-0.03	0.93	0.28	-0.10
Silique fructose	**0.75**	0.30	053	**0.90**	0.19	0.21	**0.86**	0.19	0.39	**0.83**	-0.04	0.42	0.61	0.12	0.72	0.71	0.02	0.63
Seed fructose	0.29	-0.30	**0.90**	0.26	-0.18	**0.93**	0.43	-0.29	**0.81**	0.31	-0.28	**0.86**	0.07	0.07	0.97	0.07	0.07	0.94
Silique glucose	**0.76**	0.49	0.23	0.67	0.66	0.17	**0.85**	0.34	0.34	0.67	0.52	0.45	0.60	0.16	0.72	0.67	0.05	0.64
Seed glucose	0.15	-0.26	**0.93**	0.13	-0.13	**0.97**	0.48	-0.19	**0.83**	0.28	-0.21	**0.90**	0.17	0.64	0.66	0.54	0.66	0.34
Silique sucrose	0.69	-0.69	0.17	**0.87**	-0.43	0.15	0.62	**-0.77**	0.09	0.59	-0.60	0.32	0.66	0.62	0.42	0.90	0.36	0.19
Seed sucrose	0.63	-0.67	0.25	0.68	-0.51	0.43	0.58	-0.58	0.39	0.53	-0.64	0.39	0.00	0.97	0.00	0.06	0.91	-0.40
Silique starch	-0.02	**0.84**	-0.26	0.36	**0.84**	-0.09	-0.01	0.67	-0.62	-0.22	**0.81**	-0.24	**0.78**	-0.57	-0.07	0.15	-0.94	-0.20
Seed starch	**0.78**	-0.57	0.14	**0.95**	-0.21	0.12	**0.80**	-0.47	0.25	**0.78**	-0.58	-0.10	**0.88**	0.30	0.27	0.92	0.33	0.16
Silique cellulose	**-0.91**	0.13	-0.11	**-0.90**	-0.19	-0.10	**-0.90**	0.36	-0.13	**0.93**	0.16	-0.30	**-0.91**	-0.14	-0.32	-0.93	0.21	-0.17
Seed cellulose	**-0.85**	0.30	-0.10	**-0.94**	0.07	-0.17	**-0.90**	0.36	-0.17	**0.91**	0.09	-0.15	**-0.83**	-0.34	-0.43	-0.94	0.16	-0.20

*abbreviated for factor 1 (F1), factor 2 (F2), and factor 3 (F3).

## Discussion

The close relationship between silique and seed development in canola determines the crucial function of silique in canola seed yield and quality establishment (Zhang et al. 2011; Yang et al. 2012). For example, the capacity for seed numbers in a silique and the resistance for pod shattering during seed harvest have great impacts on seed yield (Wang et al. 2007b; Gan et al. 2008). Furthermore, silique is the sole tissue that directly connects with seed through funiculus, and thus it is easy to deduce that majority of nutrients in the seed from canola plants including synthesized by silique and other green tissues or by other senescent organs, i.e. leaves, should be transported from silique (Rossato et al. 2001; Dubousset et al. 2010). Although the importance of silique on canola seed reserve formation received great attentions, detailed mechanisms from physiological, biochemical, and molecular viewpoint is scarce. Recent evidence revealed that canola seed oil content was tightly associated with *BnRBCS1A* expression levels and Rubisco activities in silique wall (Hua et al. 2012b). In another case, the expression of *BnLEC1* in canola enhanced sucrose synthesis and transport in developing seed and silique contributed to the increase of seed oil content (Tan et al. 2011). Both highlighted the molecular regulation of seed oil synthesis by silique. In this investigation, we found some distinctive differences of silique and seed weight, chlorophyll content, major carbohydrate content between HOCL and LOCL derived from RIL population over three consecutive years. 1.) HOCL had significantly higher silique and seed weight than that of LOCL. 2.) HOCL produced markedly higher chlorophyll and sugar content in silique. 3.) Larger amount of proteins accumulated in developing seed cell of LOCL while considerable higher amount of oil bodies deposited in developing seed cell of HOCL. 4.) Correlation and factor analysis revealed that silique chlorophyll and seed starch content were the two most important positive factors while silique and seed cellulose content were the two major negative factors, affecting seed oil accumulation.

Before embryo differentiation and expansion, liquid endosperm in a closure of testa (or seed coat) occupies a developing seed (Johnson-Flanagan et al. 1992). During early seed developmental stage, demand for rapid seed swelling should be met to maximize a seed volume. Consequently, HOCL and LOCL both showed high-speed expansion before embryo became dominant in a seed at around 40 DAA. In our experiments, a large amount of low molecule sugars were found at this stage (Figures 5, 6, 7), which were also reported by Morley-Smith et al. (2008). The transient accumulation of these carbohydrates might be advantageous to take up enough water for extension force in a seed and hence increment of seed fresh weight. After embryo differentiated, liquid endosperm was quickly consumed by developing embryo and it rapidly occupied the most of the seed (Hill et al. 2003; Huang et al. 2009). Marked difference of silique fresh and dry weight during embryo development and lipid accumulation between HOCL and LOCL indicated that HOCL had larger capacity for water and materials such as sugar conservation and can be timely transported into seed for volume expansion. However, water potential of silique in different developmental stages should be further investigated. Notably, silique dry matter accumulated proportionally to fresh weight while there were some differences between seed dry matter and fresh weight accumulation. Although seed fresh weight kept very little fluctuation from 35 DAA to 55 DAA in 2010 and from 40 DAA to 55 DAA in 2011 (Figure 1B), it did not necessary mean that seed reached static balance. On the contrary, we interpret that there might be a homeostasis between matter exchanges. Firstly, liquid endosperm was rapidly absorbed by growing embryo and thus the embryo fresh weight was an alternative of original liquid endosperm weight. Secondly, when seed oil was forming, fructose and glucose decreased promptly at this period during the transformation of carbohydrates into lipids (He and Wu 2009; Figures 2, 3). However, regarding the relationship between seed weight and oil content, our result suggested that they were environmentally dependent (Table 1 and its contribution to seed oil content was not part of Factor 1 according to factor analysis (Tables 4 and 5). In *Arabidopsis*, Hobbs et al. (2004) reported that seed oil content was not significantly correlated with seed weight. Similar result was also observed in rapeseed that seed oil content was not significantly correlated with seed weight (Kennedy et al. 2011). However, we still propose that higher silique and seed fresh and dry weight during seed embryo development stage in HOCL could be an important basis for obtaining high seed oil content. A lower capacity of sugars loading from silique to the seed is likely to reduce the amount of substrate for oil biosynthesis. Furthermore, high seed oil content with high seed weight is desirable for seed oil yield.

As silique and seed development, both of them accumulated high chlorophyll content (Figure 4 and Additional file 1: Figure S1). However, the former has been considered as a pivotal photosynthetic organ after flowering (Chung et al. 2006; Gammelvind et al. 1996). Thus, we correspondingly monitored developing silique chlorophyll content from 2010 to 2012. Furthermore, seed chloroplast morphology was not the same to that of silique. The area of starch grain in seed cell was larger than that in silique (Figure 3A). Previous investigation showed that sun-type chloroplast contained large starch grains while shade-type chloroplast contained no starch (Lichtenthaler et al. 1981; Lichtenthaler and Burkart 1999). Given the developing seeds was in dark or weak light condition, it is surprising that seed cell contained large starch grain. The chlorophyll content in both lines declined continuously. Significantly higher content throughout the developmental stages (Figure 4 and Additional file 1: Figure S1) in HOCL indicated its high ability to produce more photo-assimilates (Lichtenthaler et al. 1981). In *Arabidopsis*, silique also accumulates chlorophyll at very early stage (before 8 DAA) and then declined quickly, which resembled with oilseed rape (Wagstaff et al. 2009 and our result). Furthermore, during silique senescence, lots of genes involved in carbohydrate metabolism are up-regulated accompanying the decreasing chlorophyll content (Wagstaff et al. 2009). However, regardless of higher silique carbohydrate content in HOCL, it is not certain that expression of corresponding carbohydrate related genes are really higher than that in LOCL during rapeseed seed development. Interestingly, both correlation and factor analysis suggested its importance to the seed oil content (Tables 1, 2, 3, 4, 5). Thus, postponing the senescence of silique could increase seed oil production.

After carbohydrate was produced in the developing silique, function of sugar in the seed should depend on its developmental schedule. Carbohydrates were important osmotic solutes in silique and seed and were involved in the compound transformation into lipid. Despite of the importance of carbohydrate during lipid biosynthesis, some researchers noticed that the function of sugar might be magnified because the amount of sugar was far less than that of lipid (Andriotis et al. 2010). However, this debate could not weaken the role of carbohydrate in this process. Previous research work has suggested that carbohydrate content in seed was closely correlated with oil content (Focks and Benning 1998; Tan et al. 2011). Relationship between carbohydrate accumulation and lipid biosynthesis should be split into two ways: 1.) carbohydrate as initial substrate for lipid synthesis (White et al. 2000); 2.) transformation efficiency from carbohydrate to lipid involved many related enzymes (Marillia et al. 2003; Gómezi et al. 2006; Ekman et al. 2008; Wakao et al. 2008). For instance, a reduction of 80% of seed oil content was found due to a dysfunction of carbohydrate metabolism, especially for the transformation of sucrose and glucose into lipid in *wrinke 1* mutant (Focks and Benning 1998). Hence, both ways can affect seed oil content.

Among these carbohydrates, hexoses content was largely and consistently higher than that of sucrose and starch at early developmental stage both under fresh and dry weight condition in silique (Figures 5, 6, 7, 8, 9), which was in accordance with previous study (King et al. 1997). Morley-Smith et al. (2008) reported that sucrose rapidly converted into hexoses after entering into seeds at early stage and kept high ratio of hexose-to-sucrose, which could explain of our results. Fructose and glucose are the low weight molecules in cells and directly take part in the lipid synthesis in seed and other metabolic pathways in silique (Chen et al. 2009), so higher content of fructose and glucose could be quickly utilized once they entered seed from silique. Unlike fructose and glucose, sucrose was always cleaved into fructose and glucose by either invertase or sucrose synthase or both (Fu et al. 1995; Ekman et al. 2008). In this study, sucrose content at early flowering stage was higher than that at late flowering stage in both HOCL and LOCL (Figure 7). Though part of the carbohydrate in silique would be transported into seed, it should retain certain amount of carbohydrate for its own development. Thus higher carbohydrate at early flowering stage is necessary because the carbohydrate content is beneficial for osmotic potential maintaining and promoting water uptake and cell elongation (Wang et al. 2000; Lalonde et al. 2003). As a result, high content of sucrose guaranteed rapid silique elongation at early developmental stages. Generally, sucrose content was in a much low level of silique tissue as comparison with seed though HOCL was significant higher than LOCL (Figure 7). The state of low sucrose content might suggest the high frequency for sucrose cleavage. It was found that very high sucrose synthase activity was observed in *Arabidopsis* and canola silique (King et al. 1997; Fallahi et al. 2008). A possible role of sucrose cleavage by sucrose synthase was suggested for energy supply for the phloem transport activity (Fallahi et al. 2008). Nevertheless, for the purpose of sucrose cleavage, high efficiency of sucrose utilization was evident in silique tissue both in high and low oil content lines. Sucrose was also catalyzed into UDP-glucose for starch and cellulose synthesis (Geigenberger 2003; Hajirezaei et al. 2003). It was proved that starch is not the dominant carbohydrate type in silique (King et al. 1997; Andriotis et al. 2010). Furthermore, starch grains were observed in the funiculus and seed coat during early seed development of *Arabidopsis* through chemical staining in silique section suggesting silique starch might be mainly transported into seed rather for participated in other silique physiological metabolism (Fallahi et al. 2008). Alternatively, although starch had function for storage, the starch content almost disappeared during oil synthesis in canola seed, thus it was suggested that starch is preferentially involved into cell division or differentiation (Fallahi et al. 2008; Andriotis et al. 2010). However, there were also studies supporting the opinion that starch content can influence oil content directly (Fan et al. 2012, Meyer et al. 2012). For example, prohibiting from starch biosynthesis can promote the rate of oil biosynthesis in a starchless mutant *Chlamydomonas reinhardtii*, BAFJ5 (Fan et al. 2012). Interestingly, a cDNA fragment encoded ADP-Glc pyrophosphorylase, which was driven under embryo-specific promoter, was expressed in the antisense model in oilseed rape. The result showed that both of the starch and lipid content was reduced in the developing seed while no significant difference of oil content at maturation stage (Vigeolas et al. 2004). This phenomenon implied that oilseed rape embryo should have compensation way(s) to increase seed oil content finally through bypassing the reduction of starch synthesis. In our experiment, both correlation and factor analysis revealed the importance of seed starch content to seed oil content. Because hexoses mainly accumulated at seed liquid endosperm stage and sucrose was cleaved into hexoses, starch content was relatively higher among these sugars and should be the major carbon source for seed lipid deposition (Figures 5*,*6*,*7*,*8). Moreover, many starch grains were contained in the chloroplast in the seed cells, larger number of chloroplasts in the seed cells of HOCL should be a key factor to accumulate more lipids during seed development. Consequently, regardless of degradation of starch, its relatively higher content may be tightly associated with lipid synthesis especially in developing seeds of HOCL.

The close relationship between cellulose content and seed oil content indicated that reduction of cellulose content to some extent should be beneficial for improving seed oil content. Another implication is that the thinner cell wall in silique might allow more light to penetrate, thus possibly to enhance seed photosynthesis as other studies suggested seed is also an important photosynthetic organ (Eastmond et al. 1996).

## Conclusion

Taken together, our investigation clearly revealed the large variation of silique and seed physiological parameters in canola lines with different seed oil content, indicating the effect of silique physiological indexes on seed oil accumulation. In this study, we suggested that carbohydrates play crucial roles in rapid organ expansion at early seed developmental stage. After embryo differentiation, the main function of carbohydrate took part in lipid biosynthesis. One of the major distinction during seed development was the higher accumulation of oil body in HOCL while more protein accumulation in LOCL in the seed cell. Increasing silique chlorophyll and seed starch content while decreasing silique and seed cellulose content may be beneficial to increase seed oil content. Furthermore, silique physiological parameters should be adopted not only by physiologists but also by agronomists to assist canola breeding programs towards to super higher seed oil content.

## Electronic supplementary material

Additional file 1: Figure S1: Dynamic chlorophyll content in developing seed of canola high oil content line (HOCL) and low oil content line (LOCL) from 10 to 65 days after anthesis (DAA) at a 5-day interval in 2012. Each value is the mean of seeds from 15 main-inflorescence siliques of five plants from each of three replicate plots. Error bars represent standard errors. “*” at each developmental stage indicates a significant difference at 5% probability between HOCL and LOCL in both years. (TIFF 8 MB)

Below are the links to the authors’ original submitted files for images.

Authors’ original file for figure 1

Authors’ original file for figure 2

Authors’ original file for figure 3

Authors’ original file for figure 4

Authors’ original file for figure 5

Authors’ original file for figure 6

Authors’ original file for figure 7

Authors’ original file for figure 8

Authors’ original file for figure 9

Authors’ original file for figure 10

Authors’ original file for figure 11

## Abbreviations

DAA: Days after anthesis
DW: Dry weight
FW: Fresh weight
GC: Gas chromatography
HOCL: High oil content line
LOCL: Low oil content line
RILs: Recombinant Inbred Lines
TEM: Transmission electron microscopy.
