# Supplementary figures and images for: Chlorophyll and carbohydrate metabolism in developing silique and seed are prerequisite to seed oil content of Brassica napus L
Source: Bot Stud. 2014 Mar 19;55:34. doi: 10.1186/1999-3110-55-34 (PMC5432831; doi:10.1186/1999-3110-55-34)

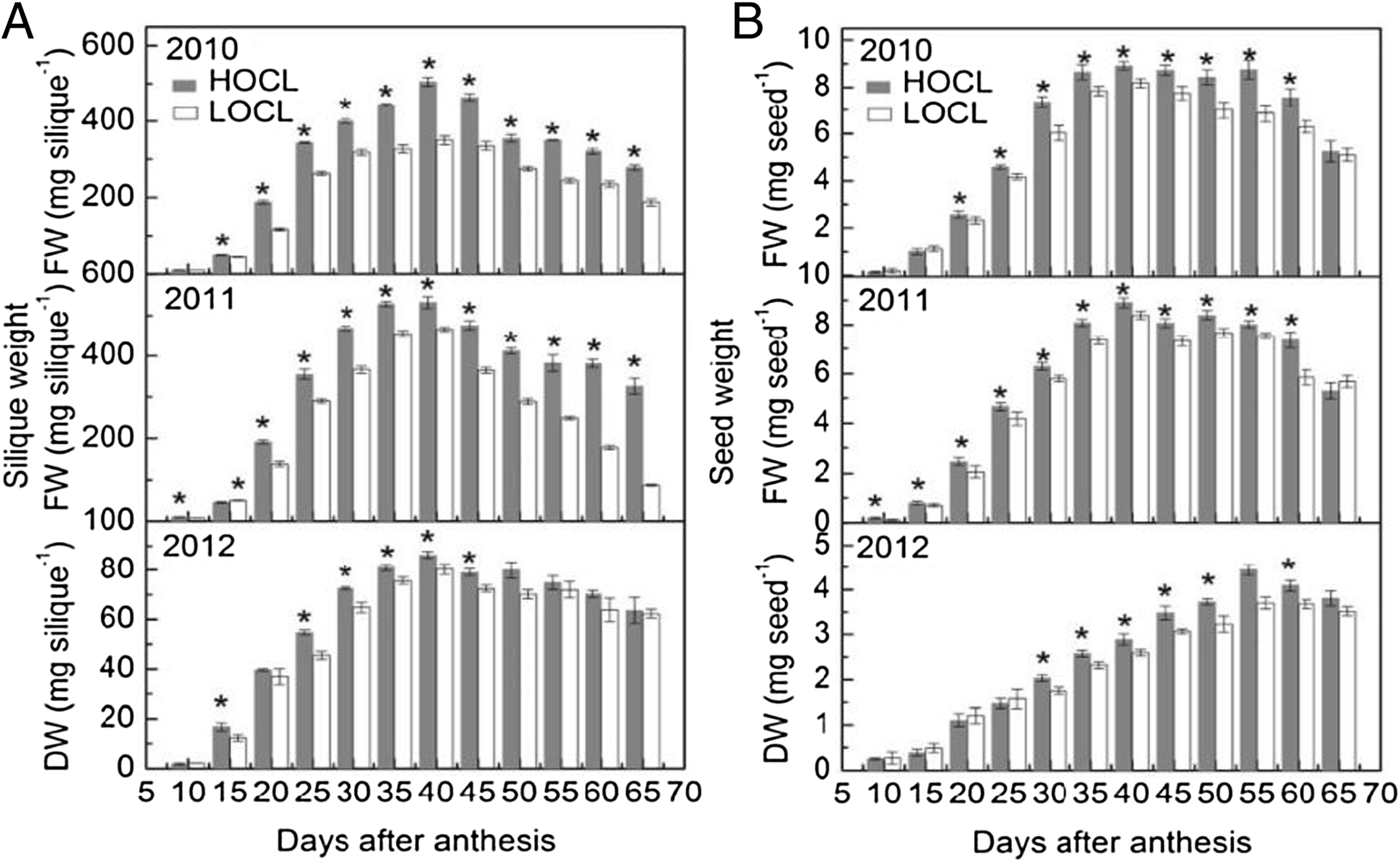

Supplement: Supplementary file 2 — Authors’ original file for figure 1 [file 40529_2013_80_MOESM2_ESM.tiff]

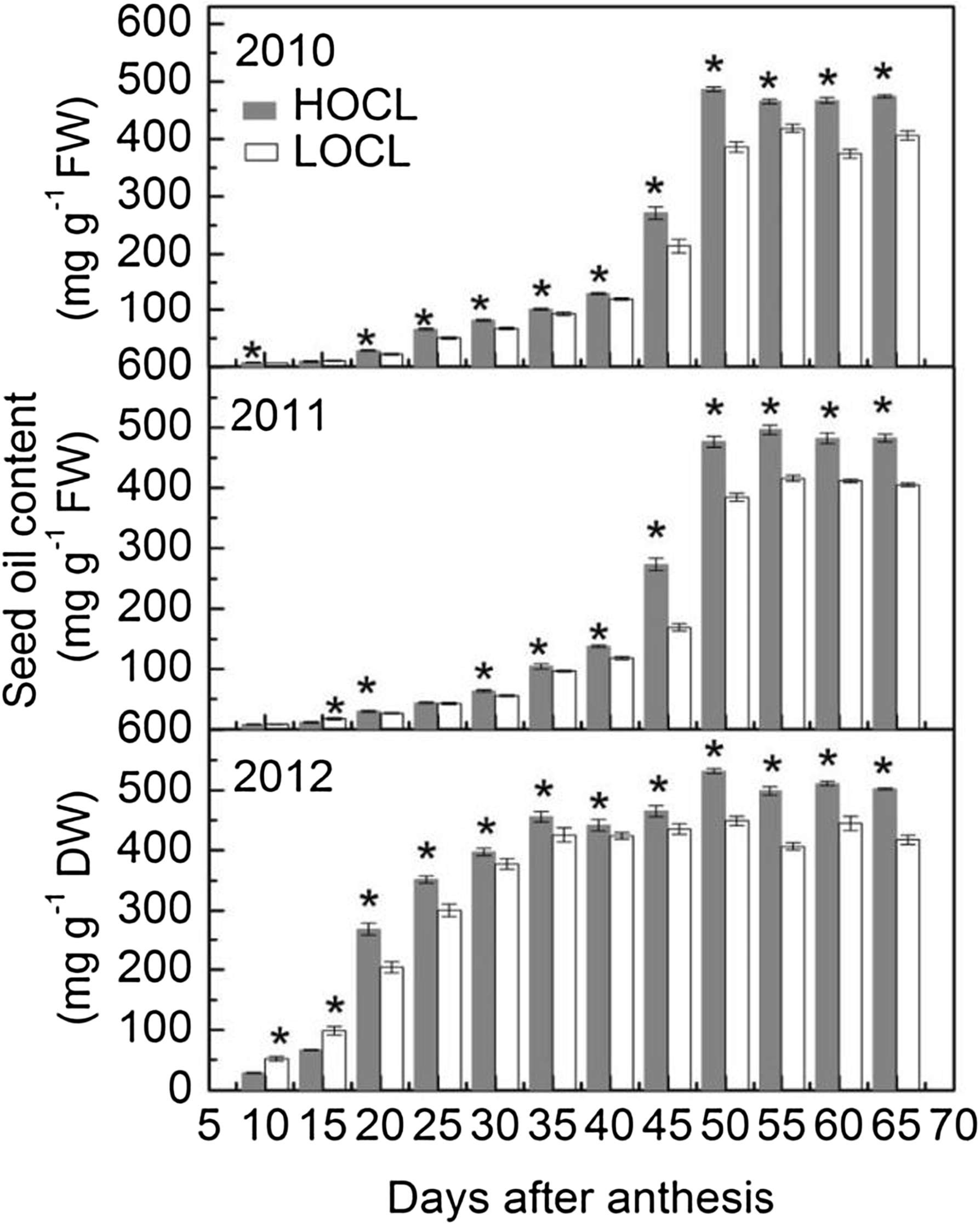

Supplement: Supplementary file 3 — Authors’ original file for figure 2 [file 40529_2013_80_MOESM3_ESM.tiff]

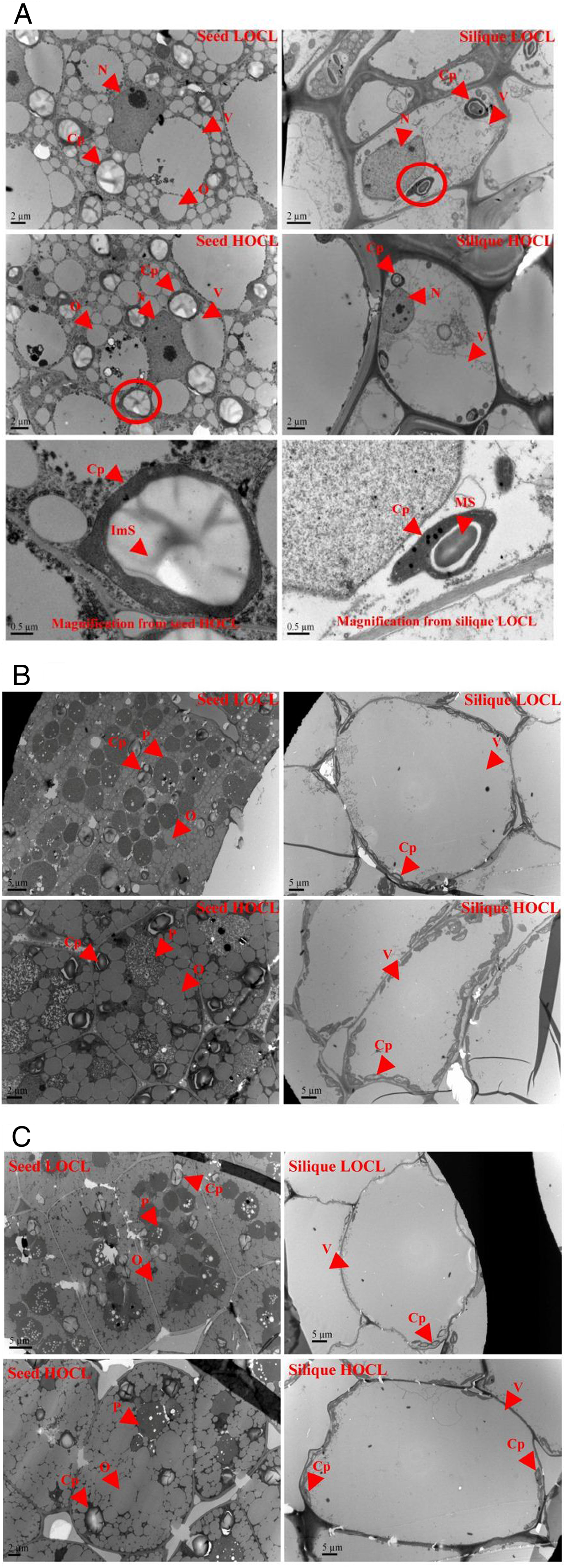

Supplement: Supplementary file 4 — Authors’ original file for figure 3 [file 40529_2013_80_MOESM4_ESM.tiff]

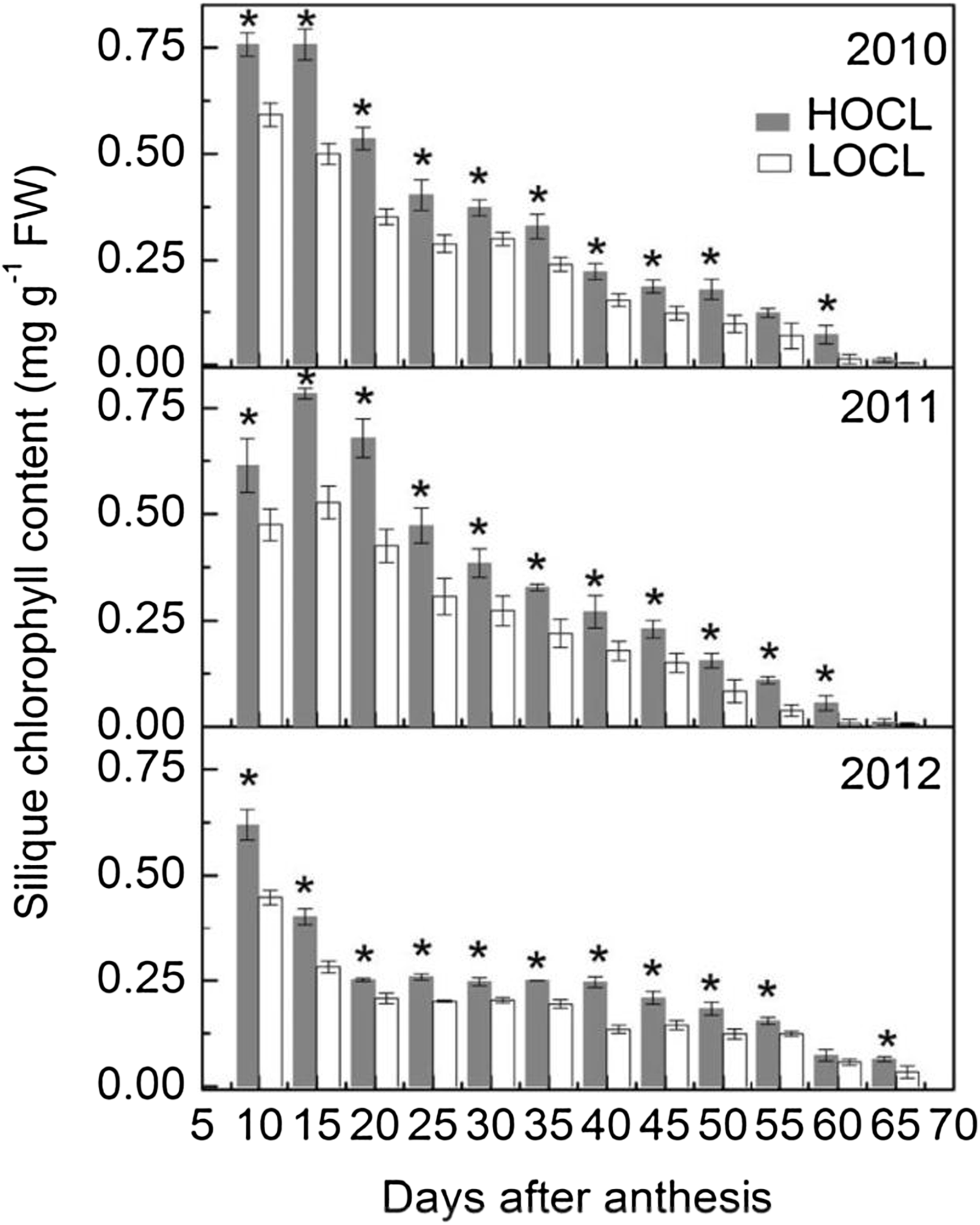

Supplement: Supplementary file 5 — Authors’ original file for figure 4 [file 40529_2013_80_MOESM5_ESM.tiff]

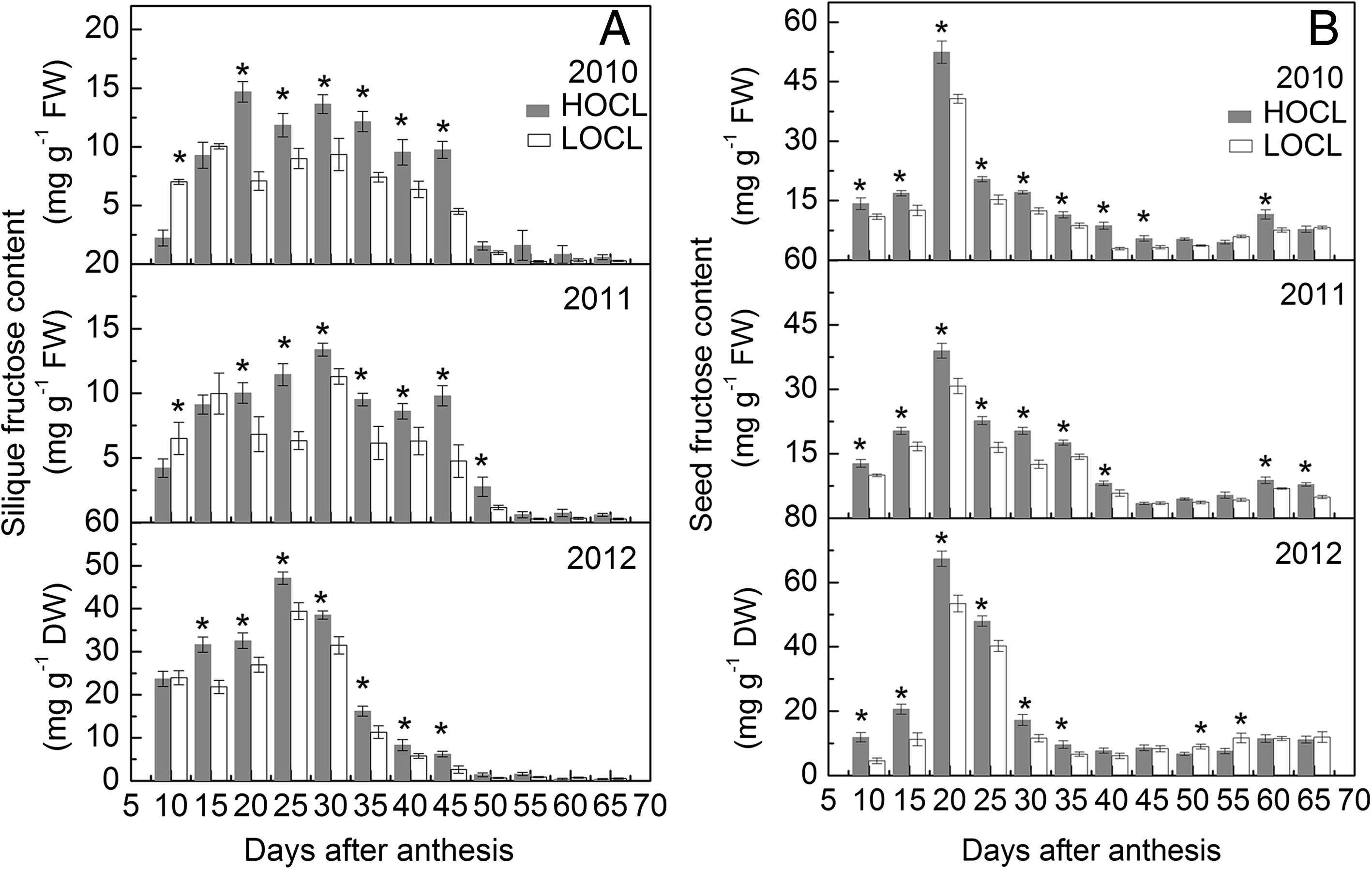

Supplement: Supplementary file 6 — Authors’ original file for figure 5 [file 40529_2013_80_MOESM6_ESM.tiff]

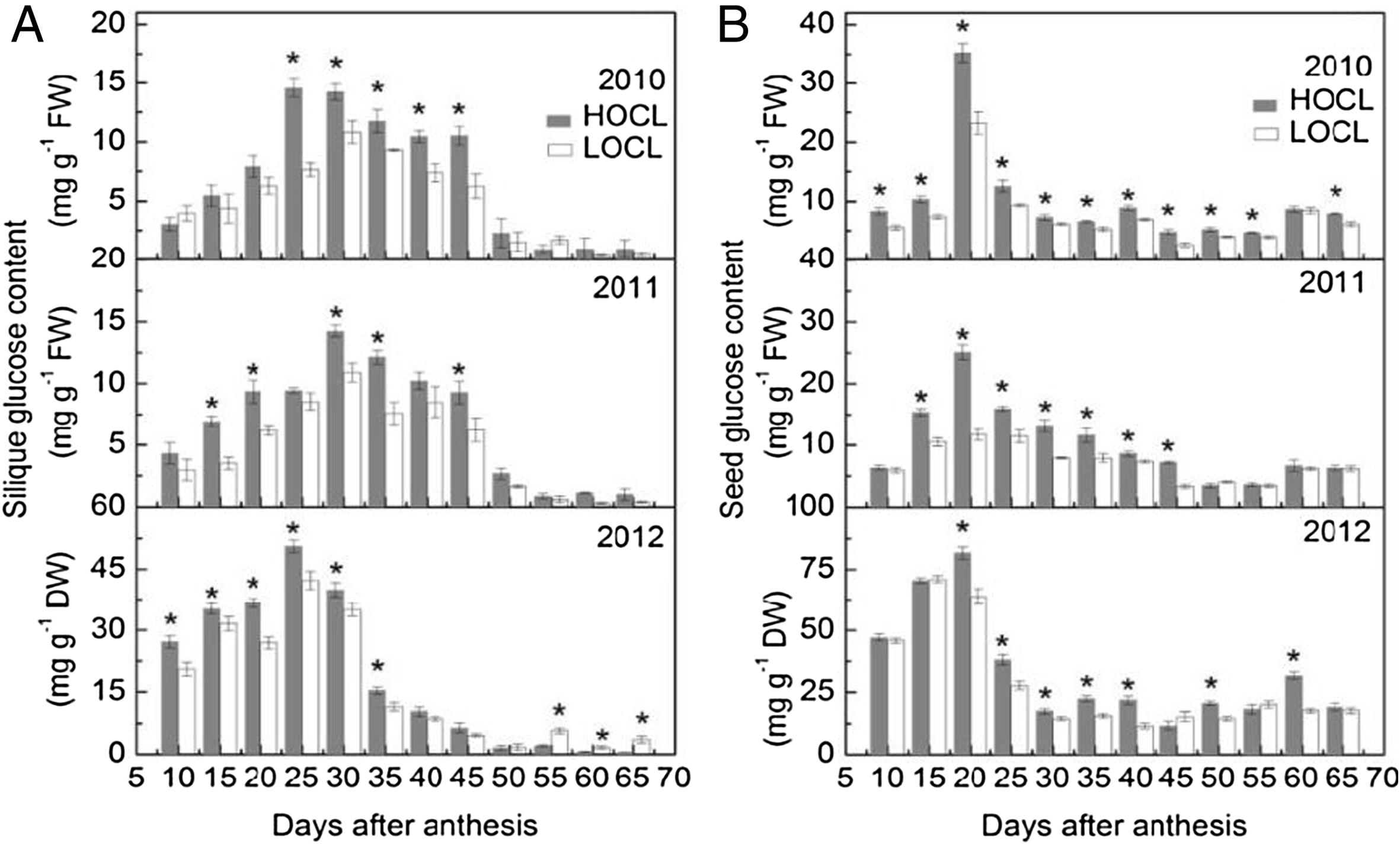

Supplement: Supplementary file 7 — Authors’ original file for figure 6 [file 40529_2013_80_MOESM7_ESM.tiff]

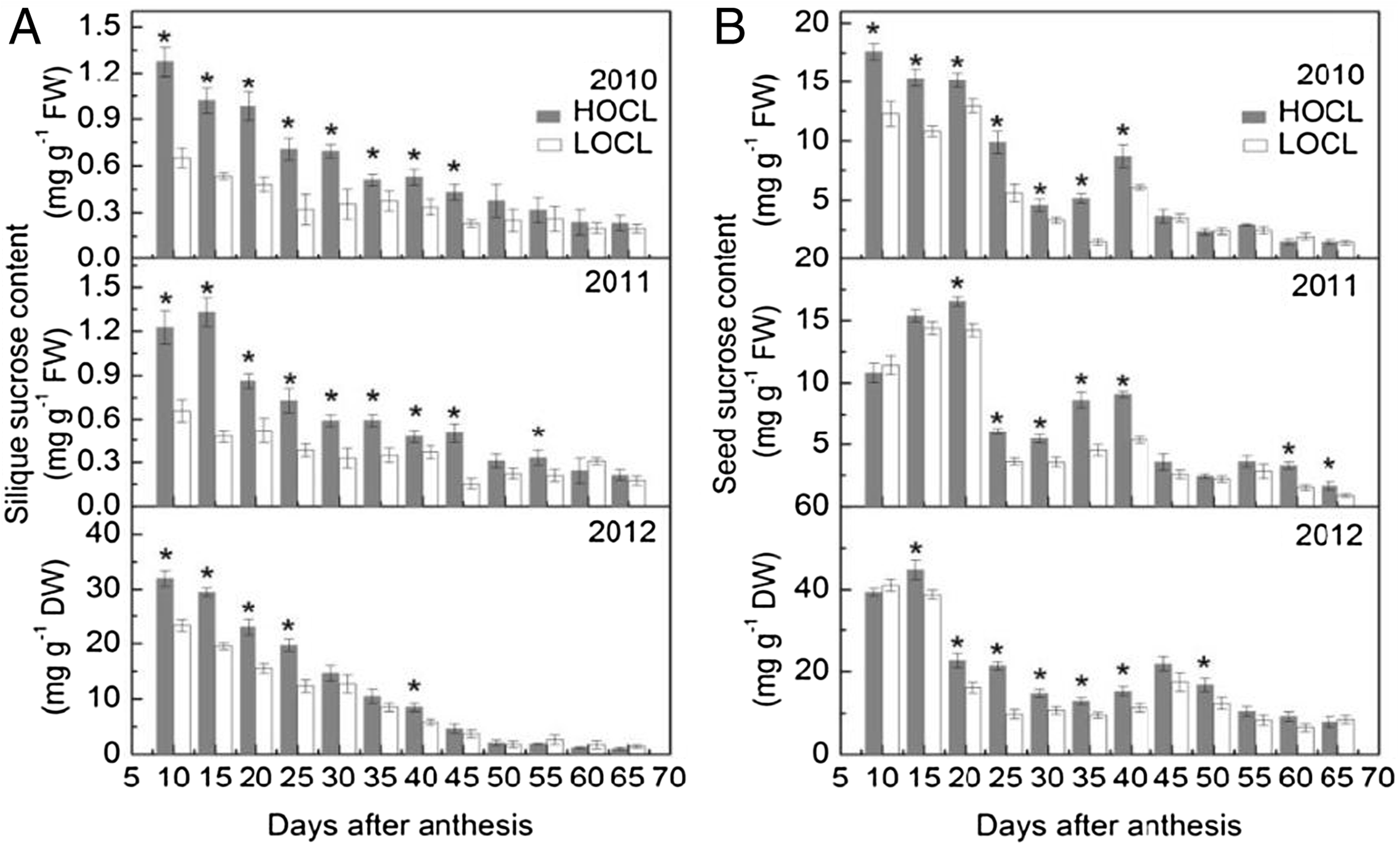

Supplement: Supplementary file 8 — Authors’ original file for figure 7 [file 40529_2013_80_MOESM8_ESM.tiff]

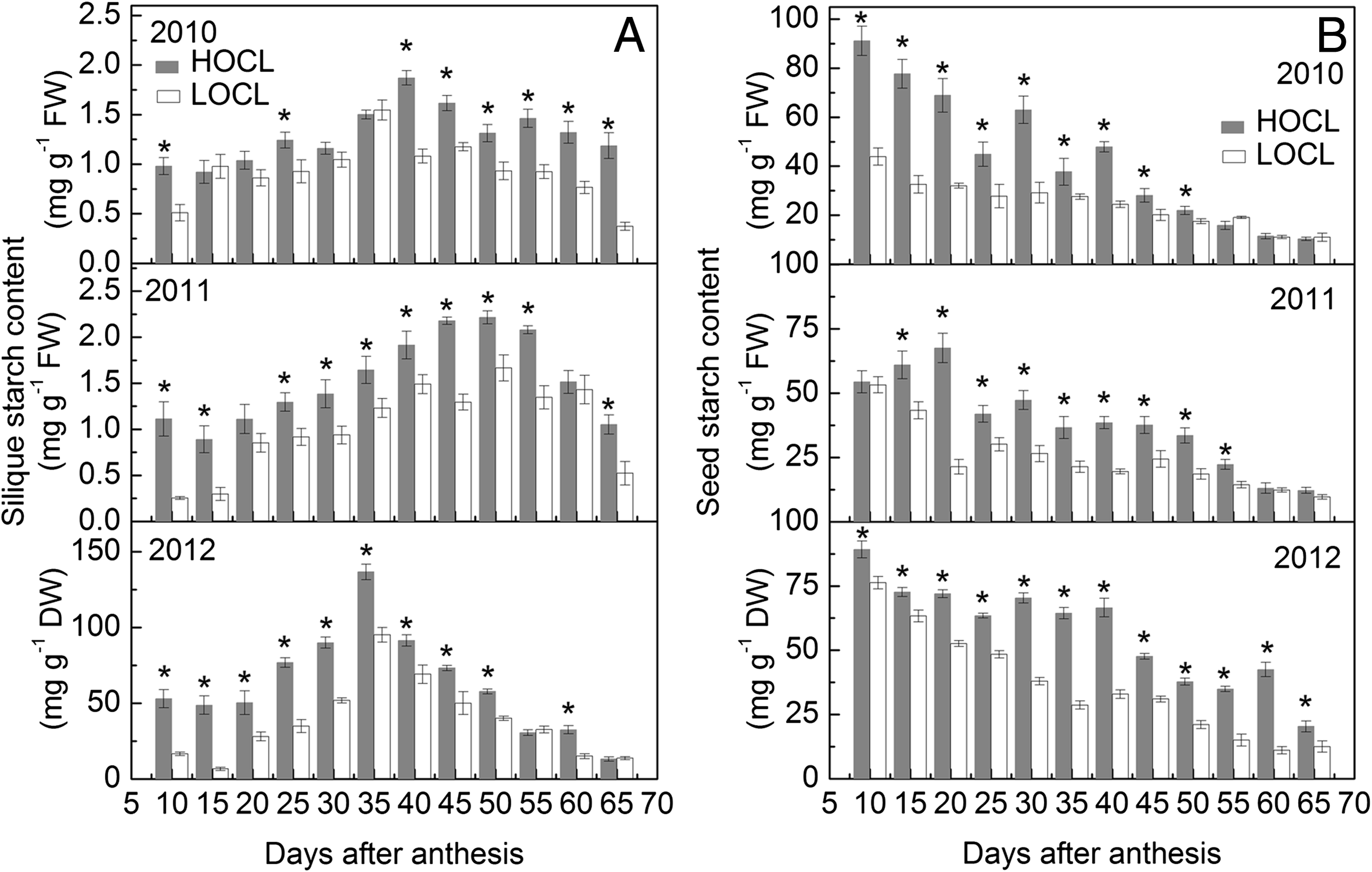

Supplement: Supplementary file 9 — Authors’ original file for figure 8 [file 40529_2013_80_MOESM9_ESM.tiff]

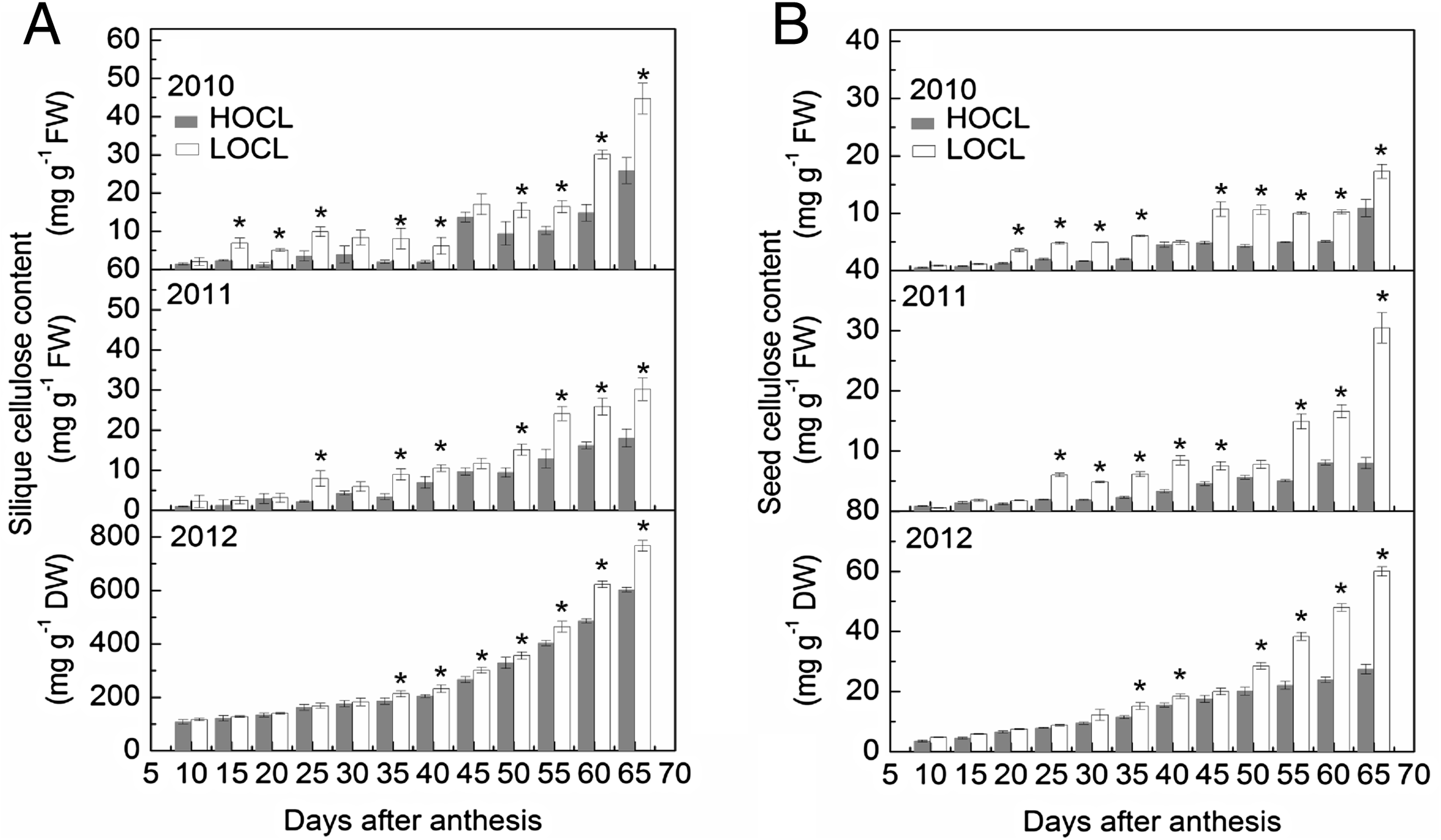

Supplement: Supplementary file 10 — Authors’ original file for figure 9 [file 40529_2013_80_MOESM10_ESM.tiff]

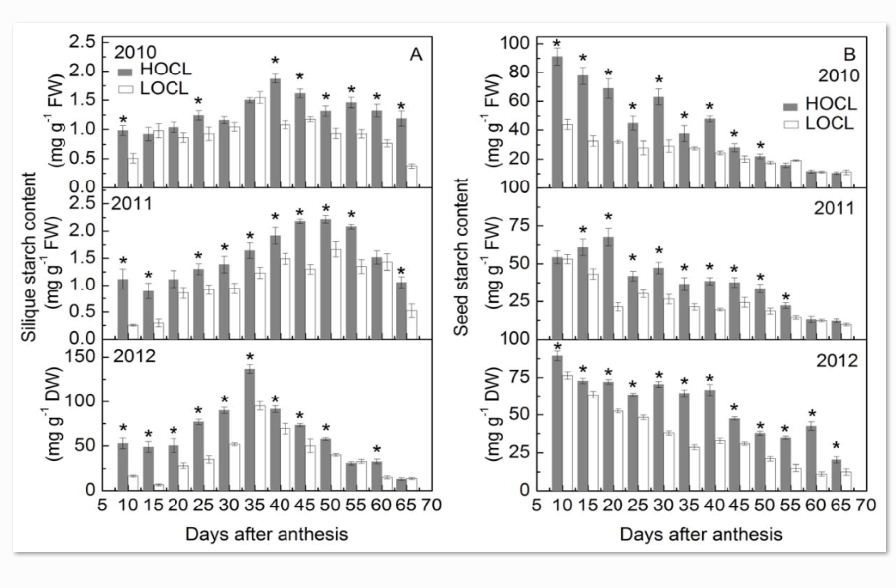

Supplement: Supplementary file 11 — Authors’ original file for figure 10 [file 40529_2013_80_MOESM11_ESM.jpeg]

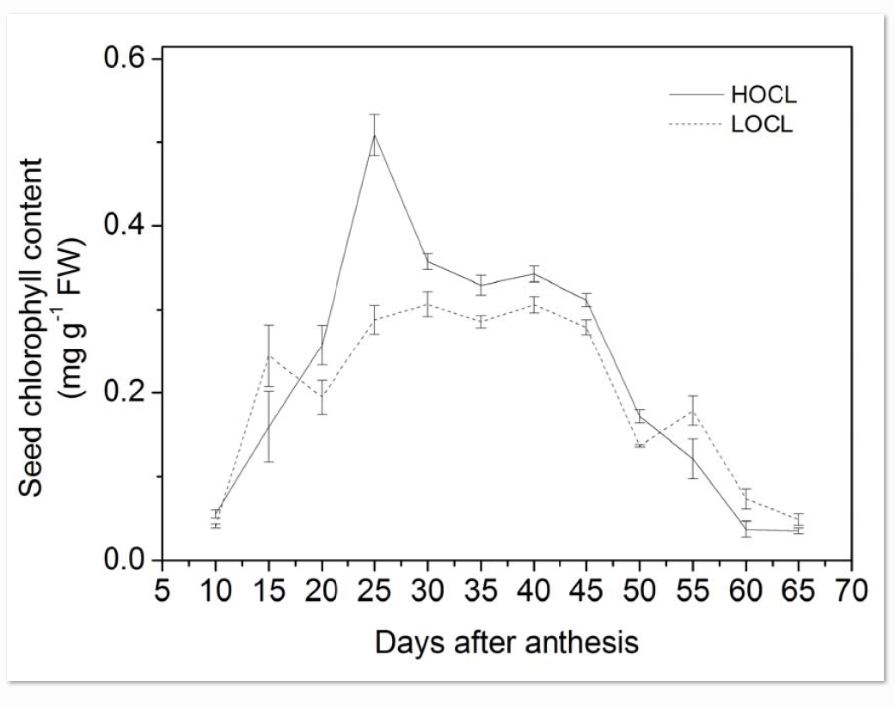

Supplement: Supplementary file 12 — Authors’ original file for figure 11 [file 40529_2013_80_MOESM12_ESM.jpeg]
